# Mouse strains to study cold-inducible beige progenitors and beige adipocyte formation and function

**DOI:** 10.1038/ncomms10184

**Published:** 2016-01-05

**Authors:** Daniel C. Berry, Yuwei Jiang, Jonathan M. Graff

**Affiliations:** 1 Division of Endocrinology, Department of Internal Medicine, The University of Texas Southwestern Medical Center, 5323 Harry Hines Boulevard, Dallas, Texas 75390-9148, USA; 2 Department of Developmental Biology, The University of Texas Southwestern Medical Center, 5323 Harry Hines Boulevard, NB5.118, Dallas, Texas 75390-9148, USA; 3 Department of Molecular Biology, The University of Texas Southwestern Medical Center, 5323 Harry Hines Boulevard, Dallas, Texas 75390-9148, USA

## Abstract

Cold temperatures induce formation of beige adipocytes, which convert glucose and fatty acids to heat, and may increase energy expenditure, reduce adiposity and lower blood glucose. This therapeutic potential is unrealized, hindered by a dearth of genetic tools to fate map, track and manipulate beige progenitors and ‘beiging'. Here we examined 12 Cre/inducible Cre mouse strains that mark adipocyte, muscle and mural lineages, three proposed beige origins. Among these mouse strains, only those that marked perivascular mural cells tracked the cold-induced beige lineage. Two SMA-based strains, SMA-Cre^ERT2^ and SMA-rtTA, fate mapped into the majority of cold-induced beige adipocytes and SMA-marked progenitors appeared essential for beiging. Disruption of the potential of the SMA-tracked progenitors to form beige adipocytes was accompanied by an inability to maintain body temperature and by hyperglycaemia. Thus, SMA-engineered mice may be useful to track and manipulate beige progenitors, beige adipocyte formation and function.

## Abstract (web summary)

Beige adipocytes are formed in response to cold and thought to contribute to organismal energy homeostasis. Here, the authors study a range of conditional and inducible RFP-expressing Cre mouse strains and find that SMA-based lines are the most useful for mapping beige adipocyte progenitor cells.

## Graphical abstract

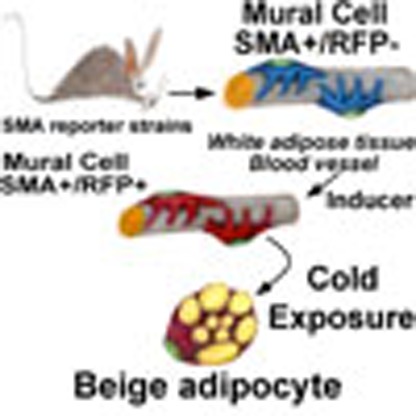

Cold temperatures and adrenergic agonists can stimulate the formation of multilocular brown adipocytes in white adipose depots^1^. These new brown adipocytes, referred to as ‘beige' or ‘brite' adipocytes, dissipate heat and consume glucose and fatty acids^2^,^3^. Because of these attributes, beige adipocytes hold therapeutic potential to combat obesity and diabetes^4^. However, the source of beige adipocytes remains controversial and transdifferentiation of unilocular white adipocytes to multilocular beige adipocytes has been a conventional notion^5^,^6^. This is exemplified in recent work of Granneman and colleagues^7^, indicating that cells marked by Adiponectin-Cre^ERT2^; red fluorescent protein (RFP), which include white adipocytes, can fate map into beige adipocytes after cold exposure. In contrast, work by Scherer and colleagues^8^ and work from others have suggested a different source; some imply a myogenic ancestry^9^,^10^. Another alternative may be smooth muscle-like progenitors that express myosin heavy chain 11 (*Myh11*), suggested by a recent cell marking/fate-mapping study by Spiegelman and colleagues^11^. Granneman and colleagues^12^ have also identified a perivascular platelet-derived growth factor receptor-α (*PDGFRα*)-positive cell as a possible source for β3 agonist-induced beige adipocytes. These studies resonate with previous proposals of a mural cell (for example, vascular smooth muscle cells and pericytes) origin for white adipose progenitors, pointing to potential commonality between white and beige adipocyte provenance^13^,^14^. Yet, Sidossis and Kajimura^15^ recently highlighted that *in lieu* of seminal fate-mapping studies, the origin(s) of beige adipocytes is not well defined and many pieces to the puzzle are missing such as age, gender and location within the adipose depot where beige adipocytes form at room temperature and after cold exposure. These obscurities are critical roadblocks to manipulating these cellular furnaces for therapeutic ends.

The Cre/loxP site-specific recombination system has provided insight into tissue development, homeostasis and function^16^. Although ‘straight' Cre drivers can be useful, they are limited for fate mapping and progenitor identification, because it is difficult to know when and where the actions of the Cre driver occurred^17^. To ascertain this information requires an extensive search, throughout development and adulthood, to delineate the temporal and spatial expression pattern of the Cre driver. Another concern of straight Cre marking is that the Cre allele can be continually expressed or functional, and also that its expression can be activated during the differentiation process of the cell type of interest, masking progenitorship^18^. Together with the other issues, this often undermines the use of straight Cre strains for fate mapping. These issues have in part been overcome by modifications that provide temporal precision, such as the tamoxifen (TM)-inducible Cre^ER/ERT2^ or the inducible/suppressible Tet systems^19^,^20^. These inducible tools are just transiently active, principally during the period in which the inducers such as doxycycline (Dox) or TM are administered^19^,^20^. During this window, Cre is active and reporter expression is turned on, presumably only in Cre-expressing cells that can be scored and sometimes in a relatively straightforward manner. After induction and chase, reporter marking can be present in potential descendants, providing insight into lineage and fate^19^,^21^. Mice that contain such inducible genetic tools have allowed scientists to identify stem cells/progenitors and to delineate the roles of these cells in tissue development, homeostasis and function^14^,^22^.

Here we examined various straight Cre (*Myf5-Cre, Myogenin-Cre and SM22-Cre*) and inducible Cre (*AdipoTrak-tTA-TRE-Cre*, *Adiponectin-Cre*^*ERT2*^, *aP2-Cre*^*ERT2*^, *Myh11-Cre*^*ERT2*^, *NG2-Cre*^*ERT2*^, *PDGFRα-Cre*^*ERT2*^, *SMA-Cre*^*ERT2*^, *SMA-rtTA-TRE-Cre* and *UCP1-Cre*^*ERT2*^) mouse strains, integrating an indelible Cre-dependent Rosa26R^RFP^ allele, to attempt to fate map beige progenitors and beiging in response to cold stimuli. We found that SMA-Cre^ERT2^- and SMA-rtTA-driven reporters and endogenous smooth muscle actin (SMA) were expressed in mural cells in all white adipose depots. Endogenous SMA expression was not detected in mature white or beige adipocytes, or other cells in the depots, and the SMA reporters mirrored that pattern at room temperature (RT) pulse. However, cold exposure triggered an evolution of SMA-dependent reporter expression and fate mapping was present in ∼60–80% of cold-induced uncoupling protein 1 (UCP1)-positive multilocular beige adipocytes in subcutaneous and visceral adipose depots. This high percentage fate mapping was supported in necessity tests; SMA mural-resident progenitors appeared essential for cold-induced beiging and the resultant mice had impaired physiological and metabolic responses to cold temperatures. SMA-based tools may therefore be useful to track and manipulate beige adipocyte development, formation and function, as well as metabolism.

## Results

### Mural cell genetic tools fate map into beige adipocytes

**Figure 1:**
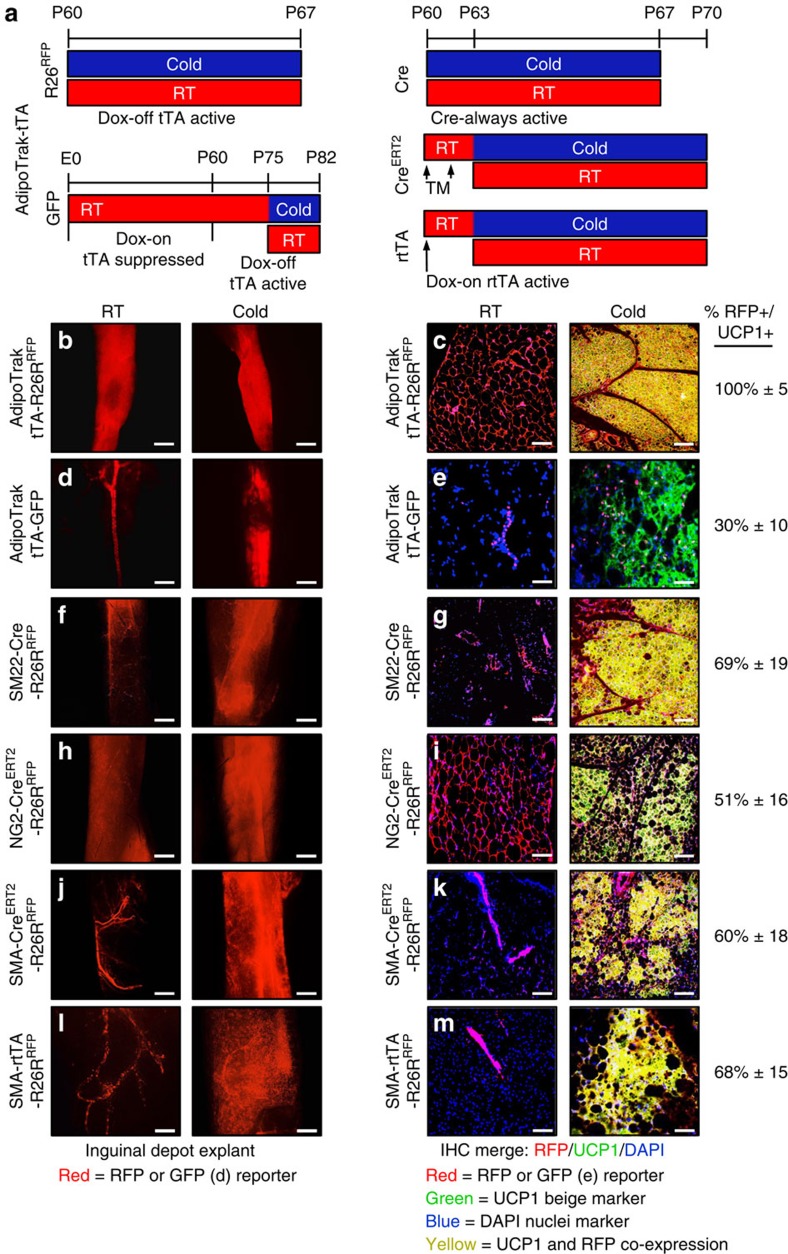
Cre drivers with high beige adipocyte labelling. (**a**–**m**) Two-month-old male Cre mice (*AdipoTrak-Cre* and *SM22-Cre*); *R26R*^*RFP*^ mouse models (**b**,**c**,**f**,**g**) were maintained at RT (23 °C) or cold exposed (6 °C) for 7 days as shown in **a**. Subcutaneous inguinal adipose depot explants were imaged for direct RFP fluorescence or sectioned and immunostained for reporter (RFP, red) and UCP1 (green); co-expression (RFP+UCP1) is a yellow-gold hue. Cell nuclei were counterstained with DAPI (blue). Two-month-old male, inducible Cre mice (*NG2-Cre*^*ERT2*^; *SMA-Cre*^*ERT2*^ and *SMA-rtTA-TRE-Cre*); *R26R*^*RFP*^ mice (**h**–**m**) were administered one dose of TM on 2 consecutive days or administered Dox 3 days before experimentation. Mice were maintained at RT or cold exposed for 7 days as shown in **a**. Subcutaneous inguinal adipose depot explants were imaged or were sectioned and immunostained for reporter (RFP, red) and UCP1 (green). For *AdipoTrak-GFP* suppression and reactivation studies, *AdipoTrak-GFP* mice were maintained on Dox from conception until 2 months of age (**a**,**d**,**e**). Dox was removed for 2 weeks and subsequently mice were maintained at RT or cold exposed for 7 days off Dox as shown in **a**. Subcutaneous inguinal adipose depot explants were imaged or sectioned and immunostained for reporter (GFP, false coloured red) and UCP1 (green) (**e**). Quantification of total UCP1+ beige adipocytes that were RFP+ is indicated to the right of the images. Images are representative from *n*=4 mice per group replicated twice. Scale bar, 10 μm (**b**,**d**,**f**,**h**,**j**,**l**). Scale bar, 100 μm (**c**,**e**,**g**,**i**,**k**,**m**).

We analysed a variety of genetically modified mice that express Cre or inducible/suppressible forms of Cre in adipose, muscle and mural cell lineages for the potential to mark cold-induced beige adipocytes in either subcutaneous inguinal or visceral perigonadal adipose depots. To visualize Cre-dependent marking, we incorporated an indelible *Rosa26R*^*RFP*^ (tdTomato) reporter allele that expresses RFP in Cre-expressing cells and in potential descendants^23^. RFP is well suited for cell-fate studies, as it is quite sensitive, can be directly visualized with fluorescence microscopy and is convenient for immunohistochemistry (IHC) and flow cytometry (quantification and isolation)^14^. We performed experiments on 2-month-old Cre- or Cre^ERT2^*-Rosa26R*^*RFP*^ marking mice that were randomly housed for 7 days in either cold temperature (6 °C), to induce the beiging phenomena, or RT (23 °C), to serve as a control (Fig. 1a). To determine whether cold exposure induced beige adipocytes derived from a Cre-marked source, we examined RFP fluorescence either from intact depots or from histological sections, the latter combined with *UCP1* IHC to help define beige adipocytes^24^,^25^. Our rational for whole depot imaging was to provide an overview of potential effects and to assess whether reporter expression might, for example, show a cold-induced evolution from vascular marking into new adipose tissue expression, as such a change may indicate that the marked cells were an origin for the beige phenomena. Beiging was less prominent in visceral perigonadal adipose depots, consistent with many scientists' observations^6^,^8^,^12^,^26^, but cold exposure did induce the formation of small islands of beige adipocytes within this depot from male mice.

To attempt to probe the adipose mural compartment and existing white adipocytes, two candidates for beige origin, we examined *AdipoTrak*; *RFP* (*PPARγ*^*tTA*^*; TRE-Cre; Rosa26R*^*RFP*^) mice^13^,^14^,^27^,^28^. The *AdipoTrak* system marks the entire white adipose lineage including mural resident progenitors and white adipocytes. Consistent with reports^13^,^14^,^27^,^28^, *AdipoTrak*; *RFP* marked the adipose-mural vasculature and apparently 100% of white unilocular adipocytes within the inguinal and perigonadal adipose depots, both in whole depot images and in histological sections at both cold temperature and RT (Fig. 1b,c and Supplementary Fig. 1a,b). In histological sections of cold-exposed *AdipoTrak; RFP* mice, 100% of *UCP1*+ multilocular beige adipocytes appeared RFP+ in inguinal and perigonadal depots (Fig. 1c and Supplementary Fig. 1b). To attempt to probe the mural compartment in a more restricted manner, we turned to *AdipoTrak; GFP* (*PPARγ*^*tTA*^*; TRE-H2B-GFP*) mice in which the green fluorescent protein (GFP) is incorporated into the chromatin of proliferating cells such as the mural cells and not into postmitotic cells such as existing white adipocytes. Nevertheless, GFP will be detected in the nuclei of adipocytes if they differentiate from a GFP-marked mural source^13^,^27^. We treated *AdipoTrak; GFP* mice with Dox, to suppress the tTA system, from conception until they reached 2 months of age. We then removed Dox for 2 weeks, to activate *H2B-GFP* in the proliferative mural progenitor cell pool and the few white adipocytes predicted to evolve from them during this time frame (Fig. 1a, lower left illustration). Consistent with that notion, in RT-housed mice some perivascular mural cells were GFP labelled and <2% of adipocytes were GFP+ (Fig. 1d,e; the black deficit present within inguinal depots is a lymph node). However, when these mice were exposed to the cold we observed that GFP+ nuclei were no longer restricted at the vasculature and now marked a much greater fraction of the adipocyte compartment, as seen in whole depot images (Fig. 1d, GFP is false coloured red in this image, to mirror the RFP colouring in Fig. 1b,f,h,j,i). IHC analyses showed that ∼30%±10 of *UCP1*+ beige adipocytes were GFP+, suggesting that some beige adipocytes emanated from this *AdipoTrak GFP*+ mural progenitor pool, which is only a subset of *AdipoTrak*+ mural cells, those that proliferated in the brief Dox-off window (Fig. 1e).

To further explore a possible mural source for beige adipocyte formation, we developed a series of mural cell Cre/inducible Cre; *Rosa26R*^*RFP*^ mouse marking strains: *SM22-Cre*, *NG2-Cre*^*ERT2*^, *SMA-Cre*^*ERT2*^ and *SMA-rtTA; TRE-Cre*^21^,^29^,^30^. We found that *SM22*- (transgelin), *NG2*- (chondroitin sulfate proteoglycan 4) and *SMA*-driven reporters marked vascular-resident mural cells in many tissues including adipose depots (Fig. 1f–m and Supplementary Fig. 1c–j), consistent with previous reports^3^,^21^,^31^,^32^,^33^. For example, *SM22-Cre*; *RFP* marked the adipose tissue (subcutaneous and visceral) vasculature at RT in whole depot and IHC images (Fig. 1f,g and Supplementary Fig. 1c,d). This pattern elaborated on cold exposure with expansion of RFP expression in whole depots and in histology with 69%±19 of inguinal and 63%±13 of perigonadal *UCP1*+ beige adipocytes co-expressing RFP (Fig. 1g and Supplementary Fig. 1d). We also observed this pattern with two inducible mural cell Cre (*NG2-Cre*^*ERT2*^ and *SMA-Cre*^*ERT2*^) mice that we randomized to either vehicle or to one dose of TM (250 mg kg^−1^ per day) for 2 consecutive days. Forty-eight hours after the last injection, we housed the mice for 7 days either at RT (23 °C) or in the cold (6 °C; Fig. 1a). We found that *NG2-Cre*^*ERT2*^; *RFP* marked some mural cells and essentially 100% of adipocytes in subcutaneous adipose depots (inguinal and periscapular; Fig. 1h,i). However, in visceral depots RFP marked only the mural vasculature and not adipocytes, providing a potential tool for the visceral location (Supplementary Fig. 1e,f). In response to the cold we observed that ∼51%±16 of subcutaneous inguinal and 49%±11 of visceral perigonadal *UCP1*+ beige adipocytes were RFP+ (Fig. 1i and Supplementary Fig. 1f). The fact that ∼100% of subcutaneous white adipocytes were labelled at RT and only ∼50% of *UCP1*+ beige cells were marked after cold exposure indicates that beige sources other than white adipocytes probably exist, the data supported by the results with the visceral beige marking outcomes. We next assessed *SMA-Cre*^*ERT2*^; *RFP* mice. At RT, RFP+ cells were present in mural vascular positions of all adipose depots but not in adipocytes of any adipose depot (Fig. 1j,k and Supplementary Fig. 1g,h, not shown). On cold stimulation, RFP expression elaborated and we found that 60%±18 of subcutaneous inguinal and 65%±15 of visceral perigonadal *UCP1*+ beige adipocytes were RFP+ (Fig. 1k and Supplementary Fig. 1g,h). We observed similar results with a *SMA-rtTA; RFP* Dox-inducible mouse strain (*SMA-rtTA; TRE-Cre; Rosa26R*^*RFP*^). Dox was administered 3 days before 7 days of RT or cold exposure (Fig. 1a). At RT, RFP was restricted to the mural vasculature compartment in subcutaneous and visceral depots (Fig. 1l,m and Supplementary Fig. 1i,j). In response to the cold, *SMA-rtTA; RFP* marked the adipocyte compartment in whole depots, and in histology the RFP reporter fate mapped into 68%±15 and 55%±19 of *UCP1*+ beige adipocytes in the inguinal and perigonadal adipose depots, respectively (Fig. 1l,m and Supplementary Fig. 1i,j).

### Cre models with low beige-adipocyte labelling

**Figure 2:**
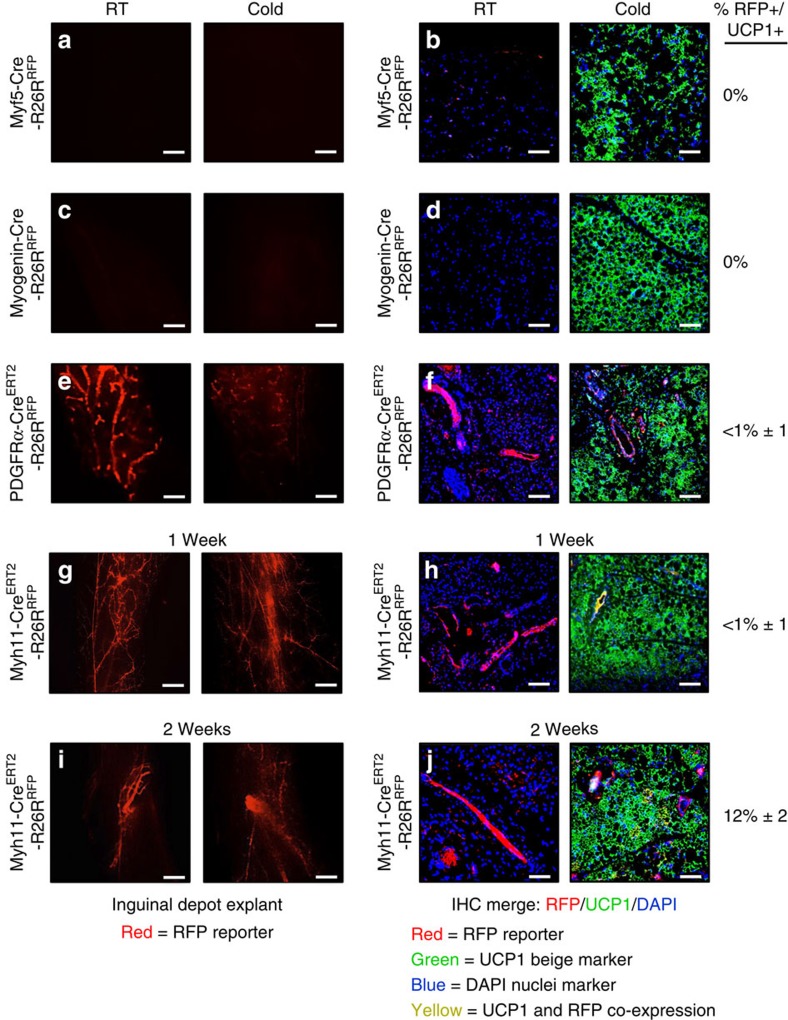
Cre models with low beige adipocyte labelling. (**a**–**h**) Two-month-old male Cre mice (*Myf5-Cre* and *Myogenin (MyoG)-Cre*); *R26R*^*RFP*^ mouse models were maintained at RT (23 °C) or cold exposed (6 °C) for 7 days. Subcutaneous inguinal adipose depot explants were imaged for direct RFP fluorescence or sectioned and immunostained for reporter (RFP, red) and UCP1 (green) (**a**–**d**). Two-month-old male, inducible Cre mice (*PDGFRα-Cre*^*ERT2*^ and *Myh11-Cre*^*ERT2*^); *R26R*^*RFP*^ mice were administered one dose of TM on 2 consecutive days. Mice were maintained at RT or cold exposed for 7 days. Subcutaneous inguinal adipose depot explants were imaged or sectioned and immunostained for reporter (RFP, red) and UCP1 (green) (**e**–**h**). (**i**,**j**) *Myh11-Cre*^*ERT2*^*; R26R*^*RFP*^ were also housed in the cold or RT for 14 days. Subcutaneous inguinal adipose depot explants were imaged or sectioned and immunostained for reporter (RFP, red) and UCP1 (green). Quantification of UCP1+ beige adipocytes that were RFP+ is denoted next to the IHC image. Images are representative from *n*=4 mice per group replicated twice. Scale bar, 10 μm (**a**,**c**,**e**,**g**,**i**). Scale bar, 100 μm (**b**,**d**,**f**,**h**,**j**).

As the muscle has been proposed as a brown adipocyte source^9^,^34^, we tested two proposed muscle (*Myf5-Cre* and *Myogenin-Cre*) Cre strains. *Myf5-Cre* is reported to mark the muscle, classical interscapular brown adipose tissue (BAT), a subset of white adipose depots and a range of other tissues^9^,^18^,^34^. *Myogenin-Cre* is reported to mark the skeletal muscle lineage^35^. We found that *Myf5-Cre; RFP* fluorescence was absent in both the subcutaneous inguinal and the visceral perigonadal adipose depots at RT and after cold exposure, consistent with previous reports^9^ (Fig. 2a,b and Supplementary Fig. 2a,b). The tool did mark adipocytes in other depots (please see below). For *Myogenin-Cre*; *RFP* mice, we did not detect RFP fluorescence in any adipose depot at either temperature (Fig. 2c,d and Supplementary Fig. 2c,d). These data are consistent with the notion that lineages marked by *Myf5-Cre* and *Myogenin-Cre* are not sources of beige adipocytes in either inguinal (a major location of beige cells) or perigonadal depots, and in the case of the skeletal muscle *Myogenin-Cre* marking strain does not appear to be sources in any of the evaluated depots.

We also tested two previously reported beige adipocyte marking inducible Cre mice (*PDGFRα-Cre*^*ERT2*^ and *Myh11-Cre*^*ERT2*^)^11^,^12^. Both *PDGFRα* and *Myh11* are reported to mark the vascular compartment of many tissues^32^,^36^,^37^. In adipose depots, *PDGFRα* marking is reported to be located in non-mural perivascular cells and to mark 45% of β3-adregeneric-induced perigonadal beige adipocytes^12^. *Myh11* is a well-described smooth muscle marker and is reported to mark a subset (15%) of cold-induced beige inguinal adipocytes^11^,^32^. We first examined *PDGFRα-Cre*^*ERT2*^; *RFP* mice and found that within adipose depots RFP was principally restricted to non-mural perivasculature positions, as previously reported^12^, at RT (Fig. 2e,f and Supplementary Fig. 2e,f). After cold exposure, RFP labelled 0–1%±1 of *UCP1*+ beige adipocytes and the few we could detect were scattered throughout the subcutaneous and visceral adipose depots (Fig. 2f and Supplementary Fig. 2f). *Myh11-Cre*^*ERT2*^; *RFP* marked the mural vascular compartment of all adipose depots at either cold temperature or RT (Fig. 2g–j and Supplementary Fig. 2g–j). After 7 days of cold exposure RFP labelled <1% of *UCP1*+ beige adipocytes but after 14 days of cold conditions ∼12%±2 and 10%±2 of inguinal and perigonadal *UCP1*+ beige adipocytes were RFP+, respectively, consistent with a previous report^11^ (Fig. 2i,j and Supplementary Fig. 2i,j). Taken together, our data suggest that beige adipocytes are not primarily from an adipocyte, a myogenic or a *PDGFRα* source, but rather from a vascular-residing mural cell source, and that mice harbouring SMA-based genetically engineered tools might be useful strains to mark and track the beige lineage.

### White adipocytes do not generate cold-induced beige adipocytes

**Figure 3:**
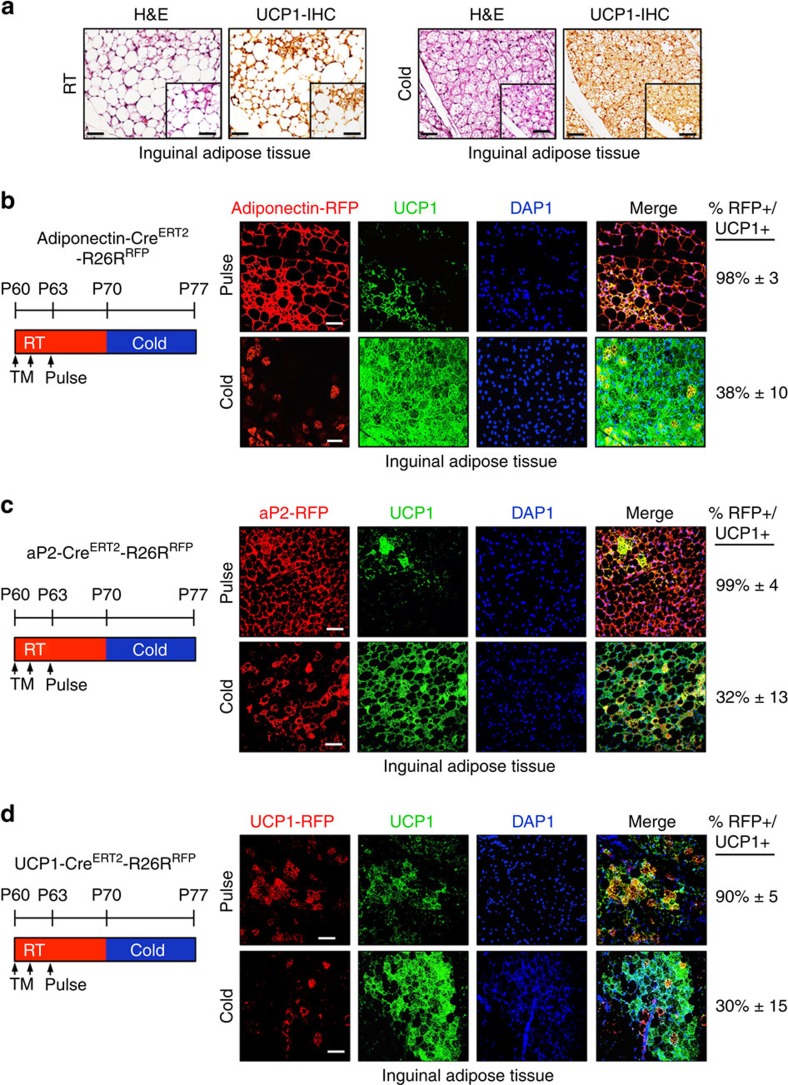
Existing white adipocytes do not contribute to beiging. (**a**) Two-month old C57BL/6J mice were maintained at RT or cold temperature for 7 days. Subcutaneous inguinal adipose depots were haematoxylin and eosin (H&E) stained and IHC stained for UCP1. Insets are magnified images. Scale bar, 100 μm (in both images). (**b**–**d**) Two-month-old *Adiponectin-Cre*^*ERT2*^*; RFP* (**b**), *aP2-Cre*^*ERT2*^*: RFP* (**c**) or *UCP1-Cre*^*ERT2*^*; RFP* (**d**) male mice were administered one dose of TM for 2 consecutive days and examined (pulse) or mice were maintained at RT for 7 days (TM washout period). Subsequently, mice were cold exposed for 7 days. Subcutaneous inguinal adipose depots were sectioned and immunostained for RFP (red) and UCP1 (green). Cell nuclei were visualized by DAPI staining. Quantification of UCP1+ beige adipocytes that were RFP+ is denoted next to the IHC image. Images are representative from *n*=3 mice per group replicated twice. Scale bar, 100 μm.

To begin to probe the adipocyte compartment in a more restricted manner, we turned to two adipocyte-marking strains, *adiponectin-Cre*^*ERT2*^ and *aP2-Cre*^*ERT2*^, and combined them with a *Rosa26R*^*RFP*^ reporter allele. We administered one dose of TM for 2 consecutive days to 2-month-old male mice. Mice were maintained at RT for 1 week and then randomized them to 1 week of either RT or cold temperature. Of note, as RT is lower than thermo-neutrality, it can stimulate beige adipocyte formation^38^,^39^ (Fig. 3a). We found that 2 days after TM administration, ‘pulse', both drivers labelled essentially all adipocytes (∼99%±5; Fig. 3b,c and Supplementary Fig. 3a,b). This included labelling of virtually all beige adipocytes that were present at RT (sub-thermo-neutral) conditions and before cold exposure (Fig. 3b,c). After cold stimulation, we found that the percentage of adipocyte driver-marked beige adipocytes dropped significantly to <40%. That is, the majority (60–70%±15) of cold-induced *UCP1*+ beige adipocytes appeared unlabelled (RFP negative) and to derive from a non-adipocyte source (white or existing beige). These percentages correspond quite closely to the percentage of beige cells labelled in the *SMA-Cre*^*ERT2*^*; RFP* or *SMA-rtTA; RFP* cold-induced fate-mapping studies (Fig. 1j–m). We also examined areas of the white depots that only contained white adipocytes, areas that lacked beige cells, for the possible presence of *UCP1* expression. We found that in both *adiponectin-Cre*^*ERT2*^; *RFP* and *aP2-Cre*^*ERT2*^; *RFP* mouse models, unilocular white adipocytes did not express *UCP1* (Supplementary Fig. 3a,b). The various amounts of adipocyte-driver-labelled (30–40%) and -unlabelled (60–70%) beige adipocytes, which do express *adiponectin* and *aP2* (ref. 8), present after cold exposure. This could be reconciled by the existence of *UCP1*+ beige cells present at RT, as ambient temperatures are below thermo-neutrality and therefore are a beiging stimulus. To further explore the pre-existing beige adipocytes and the newly formed cold-induced beige cells, we turned to an *UCP1-Cre*^*ERT2*^ mouse strain and combined the driver with the *Rosa26R*^*RFP*^ reporter allele. *UCP1* is expressed in beige adipocytes but not in white adipocytes; thus, the eponymous driver strain can differentiate between beige adipocytes that are pre-existing, and marked by the *UCP1*-driver, and perdure from white adipocytes, not labelled by the *UCP1* driver, which could undergo a cold-induced transformation into beige cells. To attempt to discriminate between beige pre-existing and white transformation, we administered one dose of TM for 2 consecutive days to *UCP1-Cre*^*ERT2*^*; RFP* P60 male mice (Fig. 3d). Mice were maintained at RT and analysed at pulse (2 days post TM induction) to visualize and score existing beige adipocytes, and to determine the recombination efficiency of the reporter compared with endogenous *UCP1* expression. We found, as reported^24^,^40^,^41^, that *UCP1*+ beige adipocytes existed in the P60 RT-housed mice, and that 90% of these *UCP1*+ beige adipocytes were RFP+, indicating a high correspondence between *UCP1-Cre*^*ERT2*^-driven reporter marking and endogenous *UCP1* expression/beige adipocytes (Fig. 3d). To attempt to estimate the percentages of beige adipocytes that perdure and those that form *de novo* after cold exposure, we TM activated the *UCP1-Cre*^*ERT2*^*; RFP* reporter to mark pre-existing beige adipocytes, waited a week and then randomized the mice to 7 days of RT or cold temperature (Fig. 3d). At RT, ∼90% of *UCP1*+ cells co-expressed the RFP reporter, just as at pulse, and cold exposure significantly reduced this to only ∼30% co-expression (Fig. 3d). This reduction from 90 to 30% roughly correlates to our fate-mapping results (60–70% marking) in which we studied cold-exposed *SMA-Cre*^*ERT2*^*; RFP* and *SMA-rtTA; RFP* mice.

### *AdipoTrak* and *Myf5-Cre* characterization

**Figure 4:**
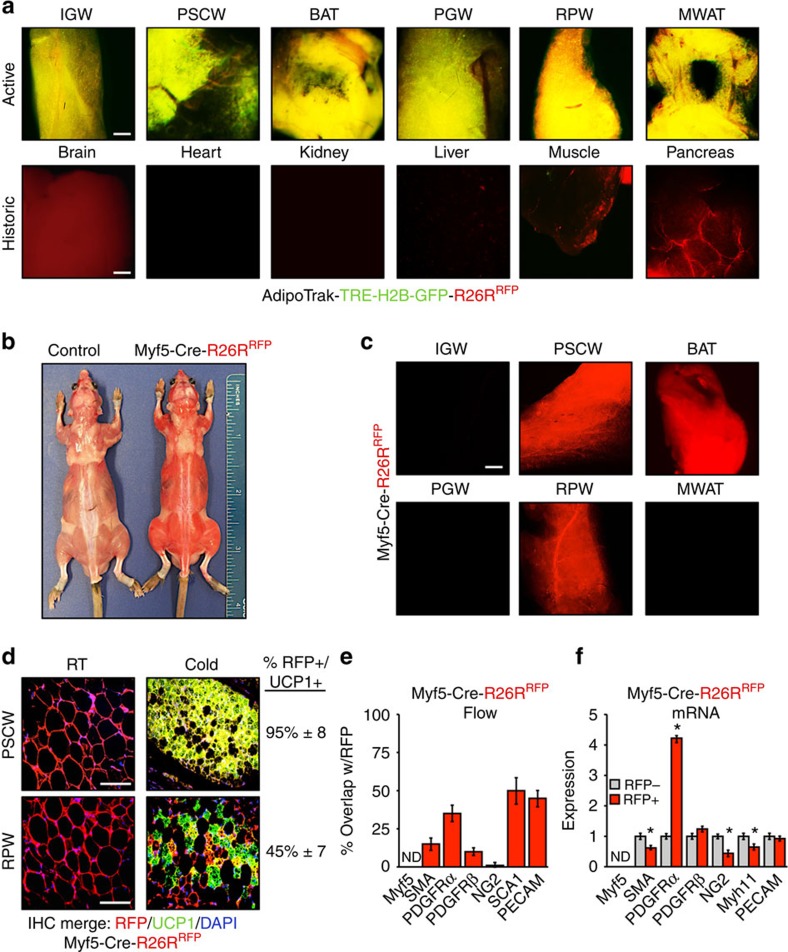
AdipoTrak and Myf5 model characterization. (**a**) Merged (*H2B-GFP* and RFP) whole-mount images of denoted adipose depots and other tissues from 2-month-old *AdipoTrak (PPARγ*^*τTA*^*,TRE-Cre); TRE-H2B-GFP; R26R*^*RFP*^ male mice and control (*PPARγ*^*tTA*^*; RFP*) mice. BAT, classical brown adipose tissue; IGW, inguinal; MWAT, mesenteric white adipose tissue; PGW, perigonadal; PSCW, periscapular; RPW, retroperitoneal. Scale bar, 10 μm. (**b**) Image of control (*R26R*^*RFP*^) and *Myf5-Cre; RFP* male mice. Please note the RFP fluorescence was easily observed in regular room lighting from the *Myf5-Cre; RFP* mouse. (**c**) Fluorescence microscopic whole-mount images of RFP fluorescence from denoted adipose depots from 2-month-old *Myf5-Cre; RFP* RT mice. Scale bar, 10 μm. (**d**) Two-month-old *Myf5-Cre; RFP* male mice were maintained at RT or cold exposed for 7 days. Histological sections from periscapular (top: PSCW) and retroperitoneal (bottom: RPW) adipose depots were examined for RFP fluorescence and *UCP1* (green) expression. Cell nuclei were counterstained with DAPI (blue). Quantification of UCP1+ beige adipocytes that were RFP+ is denoted next to the IHC image. (**e**) Adipose depot cells from 2-month-old *Myf5-Cre; RFP* mice were analysed by flow cytometry for co-expression of RFP with the indicated genes. (**f**) Adipose depot cells from 2-month-old *Myf5-Cre; RFP* male mice were separated into RFP+ and RFP− fractions using FACS and examined for mRNA expression of denoted genes using qPCR. ND, not detected. Data are means±s.e.m. (*n*=4–5 mice per group replicated twice). Scale bar, 100 μm. Student's *t*-test, *P<0.05, RFP+ compared with RFP−.

*AdipoTrak* and *Mfy5-Cre* have been exploited to examine white and brown cell biology, and here we further examined the marking potential of these mouse strains in various adipose depots and organs^9^,^13^,^27^,^28^,^34^. To further examine *AdipoTrak*, we incorporated both a *TRE-H2B-GFP* reporter and a *Rosa26R*^*RFP*^ reporter to generate a double reporter strain (*PPARγ*^*tTA*^*; TRE-Cre; TRE-H2B-GFP; Rosa26R*^*RFP*^). We combined the two marking alleles, as they provide different information: *TRE-H2B-GFP* will report active *AdipoTrak* expression and *Rosa26R*^*RFP*^ will report both active and historic *AdipoTrak* expression^13^. In this combined setting, yellow indicates both active and historic expression, whereas red indicates only historic *AdipoTrak* (*PPARγ*) expression in the absence of active expression (Supplementary Fig. 4a). We examined 2-month-old *AdipoTrak; RFP; GFP* mice and found that yellow (overlap between RFP and GFP) was only apparent in the adipose tissues (Fig. 4a). The brain, muscle and pancreas were RFP+ and lacked GFP expression, indicating that these tissues had *AdipoTrak* histories, consistent with our reports of embryonic *AdipoTrak* expression patterns^14^ (Fig. 4a). These data, along with those in Fig. 1, indicate that in adults *AdipoTrak* is principally restricted to adipose depots and may provide a tool to mark the beige progenitors and to fate map cold-induced beiging.

We next examined *Myf5-Cre* mice^42^ for RFP expression in day 17.5 embryos (E17.5) and in 2-month-old adult mice (Supplementary Fig. 4b). We found that *Myf5-Cre* E17.5 embryos and 2-month-old mice were visibly red, that is, even in the absence of fluorescent microscopy, indicative of robust fluorescence (Fig. 4b and Supplementary Fig. 4c, left panel). Fluorescence imaging of embryos showed that the dorsal region, craniofacial region, front and hind limbs, and the tail were RFP+, and the liver and the eyes were the only tissues in which we did not detect RFP (Supplementary Fig. 4c, right panel). This broad RFP expression was also observed in 2-month-old *Myf5-Cre* mice. RFP was detected in the brain, classical BAT, kidney, liver, lymph nodes, skeletal muscle, pancreas and skin (Supplementary Fig. 4d). Consistent with reports^9^,^34^,^43^, *Myf5-Cre; RFP* did not mark inguinal, perigonadal or mesenteric white adipose depots but did mark periscapular and retroperitoneal white adipose tissue and essentially all unilocular white adipocytes in these two depots appeared RFP+ (Fig. 4c,d). In the periscapular and retroperitoneal adipose depots, *Myf5-Cre; RFP* marking seemed quite indiscriminate, labelling what appeared to be essentially every cell type. For example, in adipose vessels, *Myf5-Cre; RFP* marked the endothelial cells, mural cells and other perivascular cells (Supplementary Fig. 4e, not shown). The widespread expression observed in histology was supported by flow cytometric analyses. For example, ∼55% of all stromal vascular fraction (SVF) cells were RFP+ (Supplementary Fig. 4f) and these cells encompassed mural lineages, endothelial lineages, immune cells, *PDGFRα* cells and so on. Quantitative PCR (qPCR) studies examining fluorescence-activated cell sorting (FACS)-isolated RFP− and RFP+ cells indicated that *Myf5/RFP*+ cells were not enriched in adipocyte progenitor, mural and endothelial cell markers, and in large part the RFP− and RFP+ cells had similar expression patterns (Fig. 4f). We also attempted to test whether the RFP expression in the adipose depots reflected active or historic *Myf5* expression by using both flow cytometric analyses of RFP+ cells and qPCR analyses of FACS-isolated RFP+ cells. Based on these studies the RFP+ cells did not appear to express *Myf5* protein or messenger RNA (Fig. 4e,f); that is, the RFP expression was based on historic Cre activity. We next examined whether the historic *Myf5-Cre*-driven RFP expression would mark cold-induced beige cells. Histological studies from *Myf5-Cre; RFP* mice housed in the cold showed that 95% of periscapular and 45% of retroperitoneal *UCP1*+ beige adipocytes were RFP+ (Fig. 4d). Taken together, the remarkably general marking, lack of inducibility and non-correspondence of endogenous expression to Cre-driven marking appear to be significant issues for the Myf5 stain in adipose biology and probably confound interpretation and utility.

### *SMA-Cre* ^*ERT2*^ white adipocyte characterization

**Figure 5:**
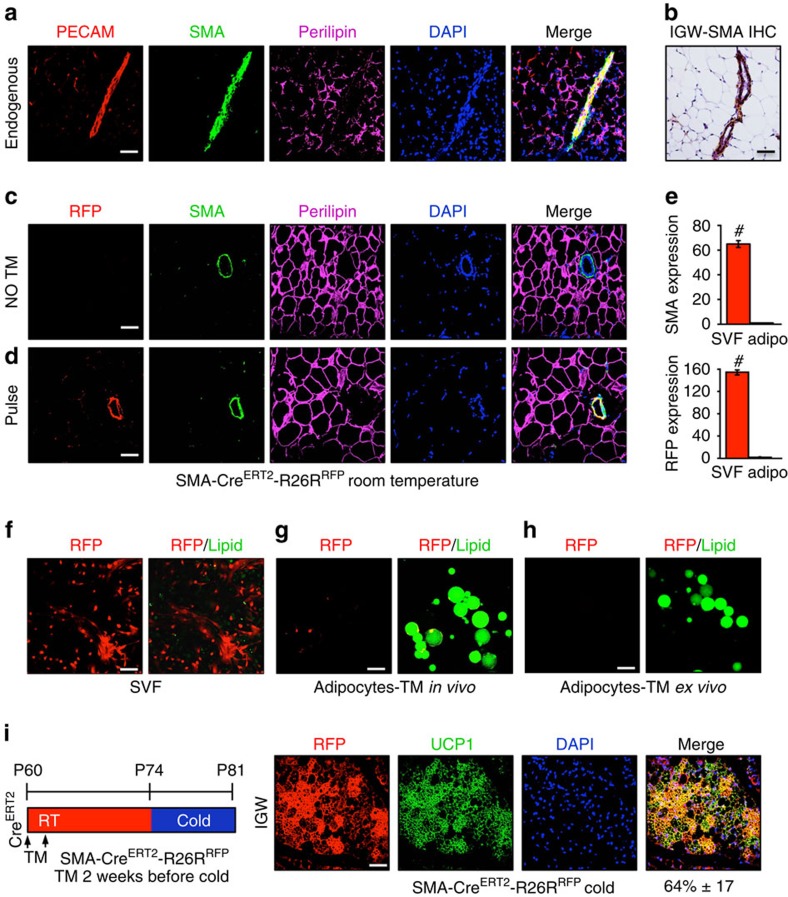
SMA-Cre^ERT2^ characterization. (**a**) Subcutaneous inguinal adipose depots from RT 2-month-old wild-type male mice were immunostained for *PECAM*, *SMA* and *perilipin*, and nuclei detected with DAPI. (**b**) Subcutaneous inguinal adipose depots from RT 2-month-old wild-type male mice were IHC stained for endogenous SMA. (**c**) Subcutaneous inguinal adipose depots from 2-month-old uninduced (no TM) *SMA-Cre*^*ERT2*^*; RFP* male mice were immunostained for RFP, SMA and perilipin, and nuclei stained with DAPI. (**d**) Subcutaneous inguinal adipose depot from 2-month-old pulsed *SMA-Cre*^*ERT2*^*; RFP* male mice were immunostained for RFP, *SMA* and *perilipin*, and nuclei highlighted with DAPI. (**e**) SV cells and adipocytes were isolated from 2-month-old TM-pulsed *SMA-Cre*^*ERT2*^*; RFP* male mice and mRNA expression of endogenous *SMA* and SMA-driven RFP were assessed by qPCR. Data are means±s.e.m. (*n*=4–5 mice per group replicated thrice). Student's *t*-test, #*P*<0.001, SVF compared with adipocyte compartment. (**f**) SV cells were isolated from 2-month-old TM-pulsed *SMA-Cre*^*ERT2*^*; RFP* male mice and visualized for RFP and lipid (green; LipidTox green). (**g**) Adipocytes were floatation isolated from 2-month-old TM-pulsed induced *SMA-Cre*^*ERT2*^*; RFP* male mice and visualized for RFP and lipid (green). (**h**) Adipocytes were isolated from 2-month-old uninduced *SMA-Cre*^*ERT2*^*; RFP* male mice. TM was then administered *ex vivo* for 24 h and the floated adipocytes examined for RFP and lipid (green). (**i**) Two-month-old *SMA-Cre*^*ERT2*^*; RFP* male mice were administered one dose of TM for 2 consecutive days. Mice were maintained at RT for 14 days (TM washout). Mice were subsequently cold exposed for 7 days. Subcutaneous inguinal adipose depots were immunostained for RFP (red) and *UCP1* (green). Cell nuclei were counterstained with DAPI (blue). Quantification of *UCP1*+ beige adipocytes that were RFP+ is denoted next to the IHC image. Images are representative from *n*=4 mice per group replicated twice. Scale bar, 100 μm.

Transdifferentiation of mature unilocular white adipocytes has been proposed as a source of beige adipocytes^5^,^6^. However, our data suggest that cells expressing a mural cell genetic signature (*SM22*+, *Myh11*+, *NG2*+ and *SMA*+) are a key source of cold-induced beige adipocytes. A possible reconciliation of the observations that the smooth muscle/mural drivers fate map into beige cells with the theorized white adipocyte transdifferentiation is that mature white adipocytes could express mural markers or marking strain-driven RFP, similar to what we observed with *NG2-Cre*^*ERT2*^ and a subset of subcutaneous white adipocytes (Fig. 1i). To explore this possibility, we further characterized endogenous *SMA* expression and *SMA-Cre*^*ERT2*^*; RFP* expression (Supplementary Fig. 5a) in white adipose depots with a focus on potential expression in adipocytes. We evaluated endogenous *SMA* expression in depots of wild-type mice using IHC. In these studies, endogenous *SMA* appeared restricted to the adipose tissue vasculature in all depots (subcutaneous and visceral) and it did not appear to be expressed in any examined mature white adipocytes (Fig. 5a,b). We also assessed endogenous *SMA* expression and *SMA-Cre*^*ERT2*^-driven RFP expression in *SMA-Cre*^*ERT2*^*; RFP* mice. In the uninduced (no TM) state, we again observed, by IHC, that endogenous *SMA* was restricted to the vasculature of all adipose depots (subcutaneous and visceral), and that endogenous *SMA* was not expressed in adipocytes (Fig. 5c). We also found that RFP fluorescence was not induced in the absence of TM (Fig. 5c). We then examined *SMA* and RFP expression in the *SMA-Cre*^*ERT2*^*; RFP* setting 2 days after administering TM. We found that endogenous *SMA* and *SMA-Cre*^*ERT2*^-driven RFP were restricted to the adipose tissue vasculature and were not expressed in adipocytes (Fig. 5d).

To further examine the possibility that *SMA* is expressed in adipocytes, we isolated both stromal vascular (SV) cells and floated adipocytes from RT-housed *SMA-Cre*^*ERT2*^*; RFP* mice 2 days after administering TM. We then quantified endogenous *SMA* and *SMA-Cre*^*ERT2*^-driven RFP mRNA expression with qPCR. We found that *SMA* and RFP mRNA appeared to be expressed only in the SV compartment and we did not detect appreciable signal in the adipocyte fraction (Fig. 5e). We next examined individual SV cells and individual adipocytes, scoring over 5,000, for RFP expression. For this, we also stained the SV cells and the floated adipocytes, and with LipidTox Green, to highlight lipids. In the fluorescent microscopic examinations, we did not detect any adipocytes that expressed RFP, unlike the positive control SV compartment (Fig. 5f,g). We performed an analogous series of studies, first isolating adipocytes and then administered TM to induce reporter expression *ex vivo* (Fig. 5h). Again, we obtained similar results: no RFP expression in adipocytes.

We next turned to cell culture studies, to examine the dynamics of *SMA* expression during white and beige adipogenesis, and to further test whether *SMA* might be expressed in cell culture-induced mature white or beige adipocytes. For this, we isolated SV cells, which contained the *SMA*-positive mural compartment (Fig. 5e), and cultured them for 7 days in white or beige adipogenic conditions and quantified endogenous *SMA* expression. We found that on either adipogenic induction *SMA* expression was rapidly and sustainably reduced (Supplementary Fig. 5c).

We also examined the possibility that TM may perdure beyond the 2-day interval after administration and activate *SMA-Cre*^*ERT2*^ in mature beige or white adipocytes during the cold exposure period. For this, we extended the washout period to 14 days, administering TM to *SMA-Cre*^*ERT2*^*; RFP* P60 mice, waiting 2 weeks before cold exposure and then examining P81 mice. We found that *SMA*-marked cells, after a longer TM washout period, still fate mapped into cold-induced beige adipocytes at roughly the same percentage as we previously observed with the 2-day interval (64%±17; Fig. 5i).

Endogenous *SMA* and *SMA-Cre*^*ERT2*^-driven RFP, before cold exposure, appeared to be restricted to the mural vascular compartment in several studies including IHC. The IHC studies also indicated that RFP and endogenous *SMA* expression had high concordance (Fig. 5d). We further examined potential concordance of endogenous *SMA* and the RFP signal using flow cytometry, to determine whether RFP-positive cells faithfully co-expressed *SMA* using cells isolated from *SMA-Cre*^*ERT2*^*; RFP* mice 2 days after TM administration (pulse). We found that virtually all RFP+ cells were *SMA*+, based on antibody staining for *SMA* (Supplementary Fig. 5d). The RFP-positive cells constituted about 50% of the total *SMA* population (Supplementary Fig. 5e). We also characterized the RFP-positive cells in flow cytometry and qPCR studies, finding that the RFP+ cells were enriched in adipocyte progenitor and mural cell markers but not in endothelial cell markers (Supplementary Fig. 5f,g). We also examined classical BAT for endogenous *SMA* expression and for *SMA-Cre*^*ERT2*^-driven RFP reporter expression. At RT or after cold exposure, both endogenous *SMA* and RFP were restricted to the vasculature and were not expressed in mature brown adipocytes (Supplementary Fig. 5h,i).

### *SMA-rtTA* white adipocyte characterization

**Figure 6:**
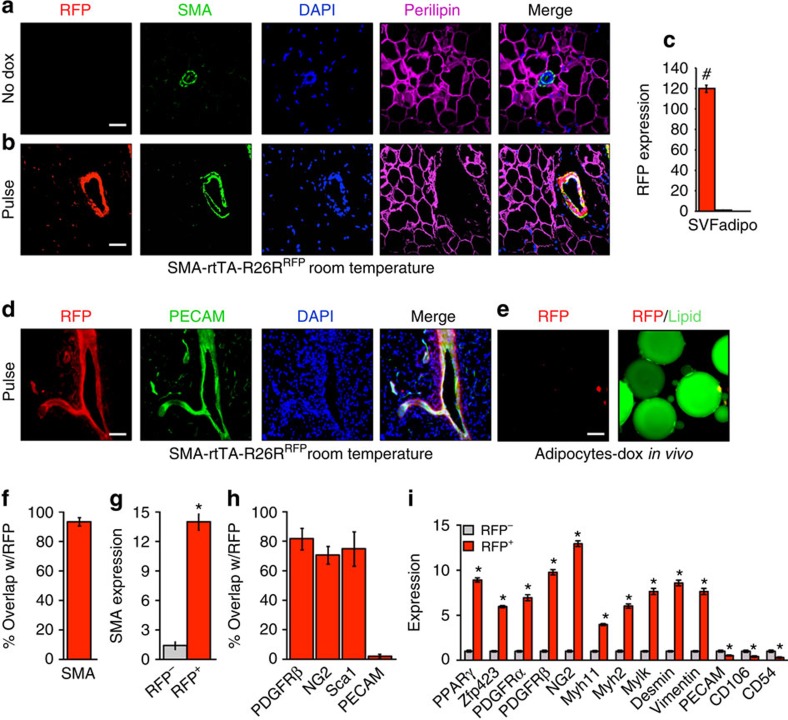
SMA-rtTA characterization. (**a**) Subcutaneous inguinal adipose depots from 2-month old uninduced (no Dox) *SMA-rtTA; RFP* (*SMA-rtTA; TRE-Cre; R26R*^*RFP*^) male mice maintained at RT were immunostained for RFP, SMA and perilipin. Cell nuclei were counterstained with DAPI (blue). (**b**) Subcutaneous inguinal adipose depot sections from 2-month old Dox-pulse *SMA-rtTA; RFP* male mice maintained at RT were immunostained for RFP, SMA and perilipin; nuclei were highlighted with DAPI. (**c**) SV cells and adipocytes were isolated from 2-month old pulse *SMA-rtTA; RFP* male mice maintained at RT. mRNA expression of RFP were assessed by qPCR. Data are means±s.e.m. (*n*=4–5 mice per group). Students *t*-test, #*P*-value <0.001, SVF compared with adipocyte compartment. (**d**) Subcutaneous inguinal adipose depots from 2-month old pulsed *SMA-rtTA; RFP* male mice maintained at RT were sectioned and immunostained for RFP and *PECAM* (green), and nuclei stained with DAPI. (**e**) Adipocytes were isolated pulse *SMA-rtTA; RFP* mice described in **d** and visualized for RFP and lipid (green). (**f**) Adipose depot cells of pulse *SMA-rtTA; RFP* mice as described in **d** were analysed for co-expression of RFP and SMA. (**g**) RFP− and RFP+ cells were FACS isolated from pulse *SMA-rtTA; RFP* mice and *SMA* mRNA expression was assessed using qPCR. (**h**) Adipose cells of pulse *SMA-rtTA; RFP* mice were flow analysed for co-expression of RFP with mural or endothelial markers. (**i**) RFP− and RFP+ cells were FACS isolated from pulse *SMA-rtTA; RFP* mice and mRNA expression of various adipose progenitor, mural and endothelial markers were assessed by qPCR. Data are means±s.e.m. (*n*=5 mice per group replicated twice). Images are representative from *n*=4 mice per group replicated twice. Scale bar, 100 μm. Students *t*-test, *P-value <0.01, RFP+ compared with RFP−.

We next examined *SMA-rtTA; RFP* (SMA-rtTA; TRE-Cre; Rosa26R^RFP^) mice (Supplementary Fig. 6a) using similar tests to those performed on the *SMA-Cre*^*ERT2*^*; RFP* mice. We first inspected *SMA-rtTA; RFP* mice in the uninduced (no Dox) state. We observed, by IHC, that endogenous *SMA* was restricted to the adipose tissue vasculature, that endogenous *SMA* was not present in adipocytes, and that RFP fluorescence was not expressed in the absence of Dox (Fig. 6a). We then examined the RFP expression pattern in adipose depots of *SMA-rtTA; RFP* mice 2 days after Dox administration (pulse). RFP was restricted to the adipose tissue vasculature and aligned with endogenous perivascular *SMA*, adjacent to *PECAM*, in all adipose depots. RFP was not visible in adipocytes (Fig. 5b,d). We also generated SV and floated adipocyte fractions from RT Dox-pulsed *SMA-rtTA; RFP* mice and quantified RFP expression with qPCR. RFP was abundantly expressed in SV cells but not in adipocytes (Fig. 6c). We also floated adipocytes after a Dox pulse and examined them for RFP expression and for lipid staining. We did not detect RFP fluorescence in any adipocytes (Fig. 6e); over 5,000 adipocytes were examined in each analysis. We next analysed RFP+ and RFP− cells with flow cytometry and found that ∼97% of RFP+ cells co-expressed *SMA* (Fig. 6f). qPCR evaluation of flow cytometry-sorted RFP+ and RFP− cells showed that endogenous *SMA* was enriched in the RFP+ cell fraction (Fig. 6g). We further examined RFP+ cells by flow cytometry and qPCR, and found that RFP+ cells were enriched in mural and adipose progenitor markers, while lacking expression of endothelial or adipocyte markers (Fig. 6h,i, not shown).

We then examined other tissues for *SMA-Cre*^*ERT2*^- and *SMA-rtTA*-driven RFP expression. We found that both models had similar tissue expression patterns (Supplementary Fig. 6b,c). In both models, RFP labelled the vasculature and smooth muscle of the brain, cardiac and skeletal muscle (although to different levels), intestine, kidney, lung, the testes and the uterus (Supplementary Fig. 6b,c, not shown). We also probed the *SMA-rtTA*-driven RFP reporter for expression in classical interscapular BAT. We found that the reporter was not expressed in mature brown adipocytes and remained at the vascular under both RT and cold conditions (Supplementary Fig. 6d). Together, the *SMA-Cre*^*ERT2*^ and *SMA-rtTA* experiments support the concordance of endogenous *SMA*, and *SMA-Cre*^*ERT2*^- and *SMA-rtTA*-driven RFP expression.

### SMA characterization in beige adipocytes

**Figure 7:**
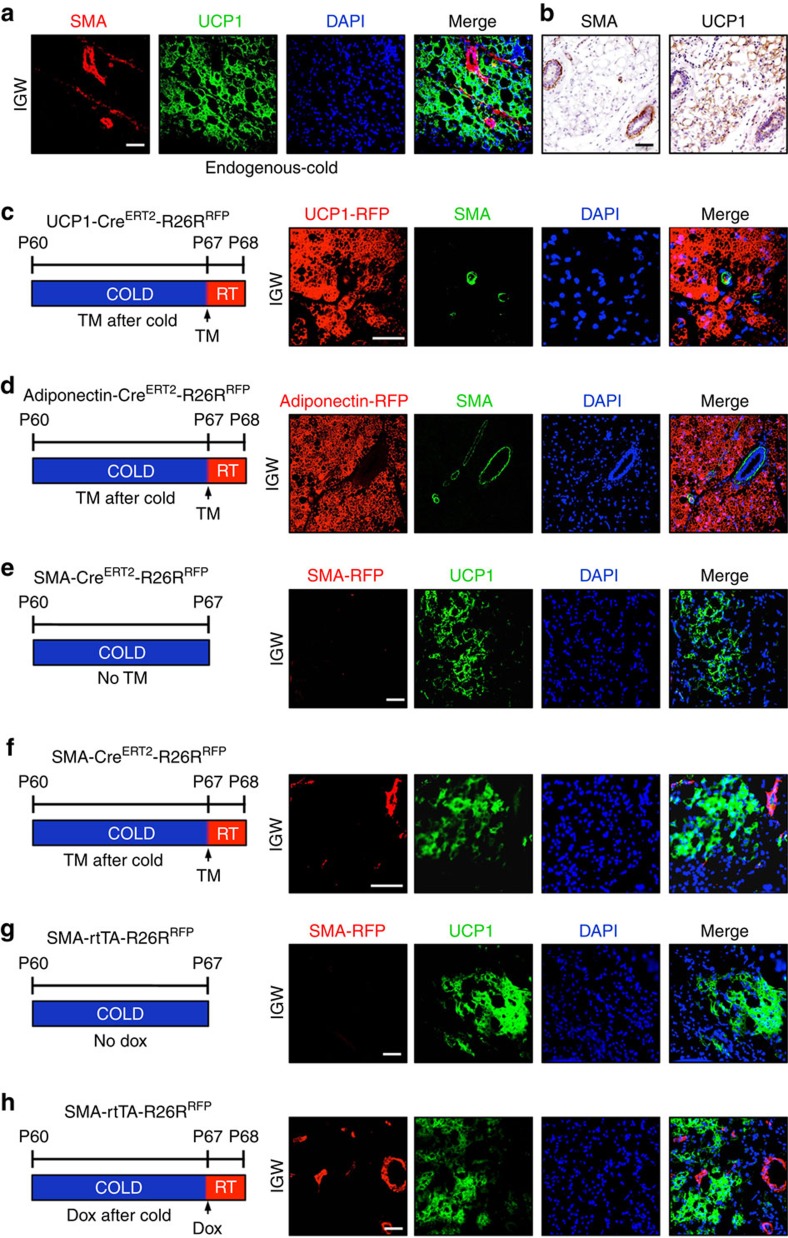
Endogenous SMA and SMA-driven reporter expression profile in beige adipocytes. (**a**) Subcutaneous adipose depots from 2-month-old, cold-exposed wild-type mice were immunostained for endogenous *SMA* and *UCP1* expression, and nuclei detected with DAPI. (**b**) Subcutaneous adipose depots from 2-month-old, cold-exposed wild-type male mice were IHC stained for endogenous SMA and *UCP1*. (**c**,**d**) Two-month-old uninduced (no TM) *UCP1-Cre*^*ERT2*^ (**c**) or *Adiponectin-Cre*^*ERT2*^ (**d**) male mice were cold exposed. After 7 days of cold exposure, mice were administered TM and maintained at RT for 24 h. Subcutaneous inguinal adipose depots were immunostained for RFP and UCP1 (green); nuclei were highlighted with DAPI. (**e**) Subcutaneous adipose depots from cold-exposed uninduced *SMA-Cre*^*ERT2*^*; RFP* were immunostained for RFP and UCP1 (green); nuclei were highlighted with DAPI. (**f**) Uninduced *SMA-Cre*^*ERT2*^*; RFP* mice were cold exposed for 7 days; mice were then pulsed with TM and housed at RT for 24 h. Sections from the subcutaneous inguinal adipose depots were immunostained for RFP and UCP1 (green). Cell nuclei were counterstained with DAPI (blue). (**g**) Uninduced *SMA-rtTA; RFP* mice were exposed to cold for 7 days. Subcutaneous inguinal adipose depot were sectioned and immunostained for RFP and UCP1 (green). Cell nuclei were counterstained with DAPI (blue). (**h**) Uninduced *SMA-rtTA; RFP* mice were cold exposed for 7 days and then administered Dox for 24 h and maintained at RT. Subcutaneous inguinal adipose depot sections were analysed for RFP (red), UCP1 (green) and DAPI (blue).

In the detailed characterizations described in Figs 5 and 6 and in Supplementary Figs 5 and 6, endogenous *SMA* expression and *SMA*-driven reporter expression appeared restricted to the mural compartment, and expression was not observed in mature white adipocytes under RT conditions. During *in vitro* beige adipogenic induction of inguinal SV cells, *SMA* mRNA expression was rapidly reduced (within 24 h) and this suppressed expression was sustained throughout the remainder of beige adipocyte differentiation (Supplementary Fig. 5c). However, it could be possible that endogenous *SMA* is expressed in white and beige adipocytes on *in vivo* cold exposure. To assess this, we housed wild-type mice in the cold for 7 days. We then examined expression of endogenous *SMA* using immunofluorescence staining and found that after cold exposure endogenous *SMA* expression remained restricted to the vasculature and was not expressed in *UCP1*+ beige adipocytes or in *UCP1*− white adipocytes (Fig. 7a,b). We continued to pursue the possibility that endogenous SMA might be expressed in white and/or beige adipocytes, by using two genetic strains to fluorescently highlight these two adipocyte cell types: *Adiponectin-Cre*^*ERT2*^; *RFP* (white and beige) and *UCP1- Cre*^*ERT2*^; *RFP* (beige). We housed uninduced (no TM) 2-month-old *UCP1-Cre*^*ERT2*^*; RFP* and *Adiponectin-Cre*^*ERT2*^*; RFP* male mice in the cold for 7 days, to stimulate *de novo* beige adipocyte formation. After cold exposure, we administered one dose of TM and maintained the mice at RT for 24 h (Fig. 7c,d). We found that *UCP1-Cre*^*ERT2*^*; RFP* marked ∼90%±7 of all *UCP1*+ immunostained beige adipocytes, indicating high correspondence between reporter and endogenous *UCP1* expression. We did not detect, by immunostaining, endogenous *SMA* expression in any *UCP1*-driven RFP-marked mature beige adipocytes. Rather, endogenous *SMA* remained restricted to the vascular compartment (Fig. 7c). We observed similar results with the *Adiponectin-Cre*^*ERT2*^*; RFP* strain. RFP labelled all (100%±6) white and beige adipocytes; however, endogenous SMA expression was restricted to the vessel and did not overlap with *Adiponectin-Cre*^*ERT2*^*; RFP* labelling (Fig. 7d).

We next used a similar series of tests to determine whether *SMA*-driven RFP expression might be induced in response to cold temperatures. For this, we examined RFP expression in cold-exposed *SMA-Cre*^*ERT2*^*; RFP* mice that had never been administered TM (Fig. 7e,f). In these uninduced cold-exposed specimens, we did not detect RFP fluorescence (Fig. 7e). We next housed 2-month old uninduced *SMA-Cre*^*ERT2*^*; RFP* male mice in the cold and after a week we induced reporter expression with a dose of TM. When induced after the cold, RFP expression appeared restricted to the blood vessels and we did not detect RFP expression in *UCP1*+ beige adipocytes (Fig. 7f). We next repeated these studies using cold-exposed *SMA-rtTA; RFP* mice that were never administered Dox or were administered Dox after a week of cold (Fig. 7g,h). In the uninduced (no Dox) specimens, we did not detect RFP fluorescence (Fig. 7g). When the mice received Dox induction after cold exposure, RFP fluorescence appeared restricted to the vasculature and was not detected in *UCP1*+ beige adipocytes (Fig. 7h). The various approaches and methods indicate that endogenous *SMA*, *SMA-Cre*^*ERT2*^*-*driven RFP and *SMA-rtTA-*driven RFP are not expressed in white or beige adipocytes. Rather, they appear to be present in the vascular compartment, although RFP does fate map into *de novo* beige adipocytes on cold exposure.

### *SMA* and *Myh11* mark different mural cell compartments

The *SM22-Cre* driver, both *SMA* drivers and the *Myh11-Cre*^*ERT2*^ driver marked the perivascular compartment and, in a restricted manner, with no apparent marking of adipocytes (Figs 1 and 2 and Supplementary Figs 1 and 2). All four of these marking strains marked cold-induced beige cells, albeit with different frequencies: *SM22* and the two inducible *SMA* drivers did so at a similarly high percentage (60–70%), whereas the inducible *Myh11* driver did so at a low percentage (Figs 1 and 2 and Supplementary Figs 1 and 2). This discrepancy could be accounted for if the various marking strains drive RFP expression in different cell types. To assess this notion, we immunostained histological samples derived from P60 *SM22-Cre; RFP* adipose depots with antibodies directed against *SMA* and *Myh11*. We found a fairly high concordance of expression of endogenous *SMA* with *SM22*-RFP. However, endogenous *Myh11* staining overlapped with *SM22*-RFP less frequently (Supplementary Fig. 7a,b). Flow cytometric studies, using antibodies directed against *SMA*, *NG2*, *Myh11* and *PECAM* endothelial marker, also indicated a diversity of marking: ∼55% of *SM22-RFP* cells co-expressed *SMA*, ∼40% of *SM22-RFP* cells co-expressed *Myh11* and ∼15% of *SM22-RFP* cells co-expressed *NG2*; co-expression with *PECAM* was minimal (Supplementary Fig. 7c). We also FACS-isolated *SM22-RFP+* and *SM22-RFP*− cells and quantified the levels of expression in the isolated cells for expression of *SMA* and *Myh11*, of other mural cell markers and of endothelial markers. We found that *SM22* and *SMA* were quite enriched in the *SM22-RFP*+ cells, and that although *Myh11* levels were higher in the *SM22-RFP*+ compared with *SM22-RFP*− cells, the enrichment was to a lesser extent (Supplementary Fig. 7d). *NG2* had roughly equal expression in *SM22-RFP*+ and *SM22-RFP*− cells, *PDGFRβ* enrichment was closer to the *SMA* patterns and *PECAM* expression was higher in the *SM22-RFP*− cells.

We next examined and compared inguinal adipose depots for endogenous expression of *Myh11* and *SMA*. We found that *Myh11* and *SMA* expression overlapped on some vessels, whereas in other vessels there was no co-expression (Supplementary Fig. 7e). This heterogeneous pattern was recapitulated in TM-pulsed *Myh11-Cre*^*ERT2*^*; RFP* mice, in comparisons of RFP and endogenous *SMA* expression. We found that *Myh11-RFP*+ cells marked the vasculature of the inguinal adipose depots, and that only some vessels showed co-localization of *Myh11-RFP* and *SMA* (Supplementary Fig. 7f). We also flow cytometrically profiled the *Myh11-Cre*^*ERT2*^*; RFP* cells. We found that ∼98% of *Myh11-RFP*+ cells co-expressed endogenous *Myh11*, and that about ∼60% of the total number of immunostained *Myh11* cells expressed *Myh11-Cre*^*ERT2*^-driven RFP (Supplementary Fig. 7g,h). The flow cytometry studies also supported the diversity of *Myh11*, *SMA* expression: ∼35% of the *Myh11-RFP* cells co-expressed *Myh11* and ∼60% of *Myh11-RFP* cells immunostained with *SMA*, whereas only ∼25% of the antibody-positive *SMA* cells co-expressed RFP (Supplementary Fig. 7i,j). We also examined the expression levels of the various mural markers in FACS-isolated *Myh11-Cre*^*ERT2*^*; RFP*+ and RFP− cells. We found that *Myh11* was highly enriched in the *Myh11-RFP*+ cells; both *SMA* and *NG2* were enriched, but to a lesser extent (Supplementary Fig. 7i,k). Together, the IHC, flow cytometry and qPCR studies indicate that there is only partial overlap between several mural cell markers, and that, similar to what is observed in other tissues and organs^21^,^29^,^30^,^31^,^42^, there is a significant mural cell and vascular cell heterogeneity within adipose depots, potentially accounting for the different percentage of fate-mapping results we observed with the different mural cell-driver mouse strains. Elucidation of the biological basis of this heterogeneity may help clarify why the inducible *SMA*-driven tools appear to have the best characteristics for analysing beige biology.

### *SMA-Cre* ^*ERT2*^-expressing cells are necessary for beiging

**Figure 8:**
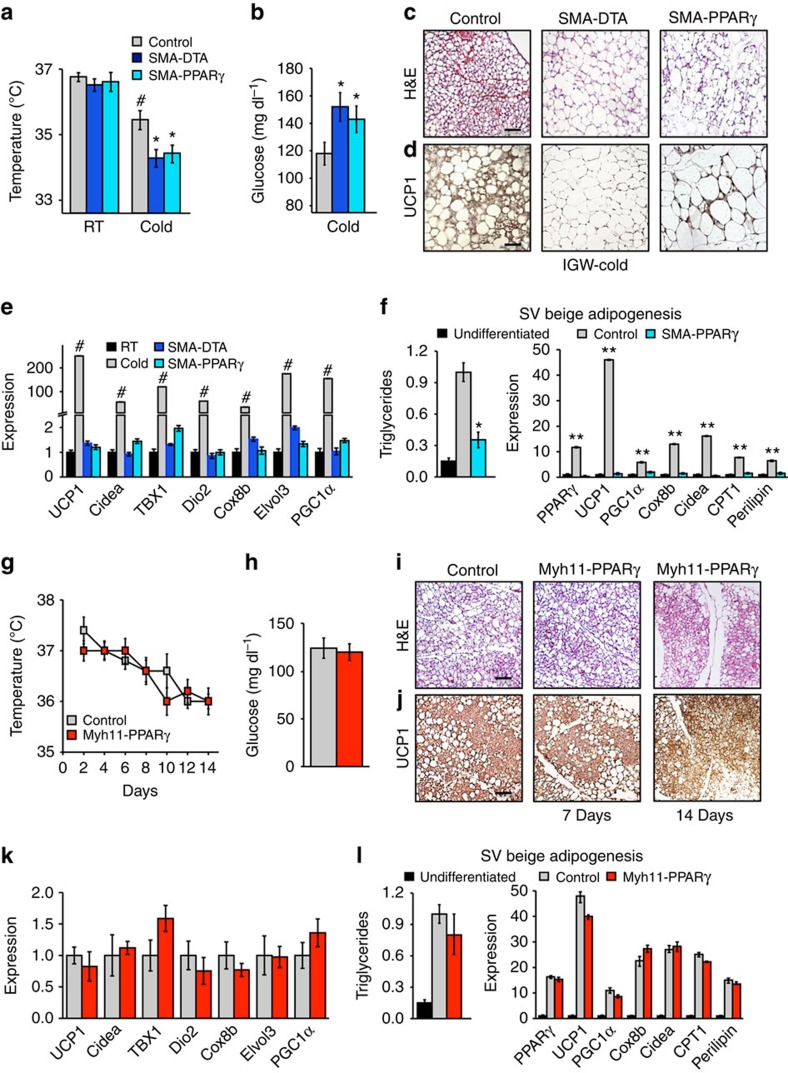
SMA+ mural cells are required for beige adipocyte formation. (**a**–**d**) Two-month-old *SMA-Cre*^*ERT2*^*; PPARγ*^*fl/fl*^ or *SMA-Cre*^*ERT2*^*; R26R*^*DTA*^ were administered one dose of TM for 2 consecutive days. Mice were then randomized to RT or cold exposed. Seven days later, mice were analysed for beige adipocyte formation by the following: rectal temperature (**a**), blood glucose levels (**b**), haematoxylin and eosin (H&E) staining (**c**), UCP1 IHC (**d**) and mRNA expression of beige and thermogenic markers (**e**). (**f**) SV cells were isolated from TM-induced S*MA-Cre*^*ERT2*^ or S*MA-Cre*^*ERT2*^*; PPARγ*^*fl/fl*^ mice and incubated in beige adipogenic culture conditions. Triglyceride content and mRNA expression of beige and thermogenic genes were analysed to determine adipocyte differentiation. Data are means±s.e.m. (*n*=4 mice per group replicated thrice). Students *t*-test, *P-value <0.05, S*MA-Cre*^*ERT2*^*; PPARγ*^*fl/fl*^ or *SMA-Cre*^*ERT2*^*; R26R*^*DTA*^ compared with control. Students *t*-test, #*P*-value <0.05, cold control compared with RT control. Students *t*-test, **P-value <0.01, control compared with undifferentiated SV cells. (**g**–**k**) Two-month-old Myh11-PPAR*γ* male mice were administered one dose of TM for 2 consecutive days. Mice were then randomized to RT or cold for 1 or 2 weeks. Mice were analysed for beige adipocyte formation by the following: rectal temperature (**g**), blood glucose levels (**h**), H&E staining (**i**), UCP1 IHC (**j**) and mRNA expression of beige markers (**k**). Data are means±s.e.m. (*n*=4 mice per group replicated thrice. Scale bar, 100 μm. (**l**) SV cells were isolated from TM-induced Myh11Cre^ERT2^; PPAR*γ*^fl/fl^ mice and incubated in adipogenic conditions. Triglyceride content and mRNA expression of beige and thermogenic genes were analysed to determine adipocyte differentiation. Data are means±s.e.m. (*n*=4 mice per group replicated twice).

To test potential necessity of *SMA-Cre*^*ERT2*^-expressing cells for cold-induced beiging, we attempted two strategies as follows: (1) a cell-killing strategy (diphtheria toxin fragment A (*DTA*))^44^ and (2) a blockade of adipocyte differentiation (*PPARγ* deletion)^45^,^46^. The *PPARγ* conditional deletion experiments were designed to test the requirement of *SMA* mural cells to generate beige adipocytes, by blocking their ability to differentiate, not the essential role of *PPARγ* in adipogenesis^47^. A concern of these approaches is that the *SMA* tools are expressed in mural compartments of many tissues and organs, and also mark other muscle types (Supplementary Fig. 6c,d)^21^. In addition, it also seems likely to be that even in adipose depots SMA is expressed in many mural cells that do not possess progenitor potential. Thus, non-autonomous issues may cloud the data, as many cellular debris created by the DTA cell-killing strategy. To attempt to minimize some of these concerns, the studies were conducted in a temporally regulated manner and proximate to cold exposure (Supplementary Fig. 8a). For these studies, we integrated conditional *Rosa26R-DTA* or conditional *PPARγ*^*fl/fl*^ alleles with the inducible *SMA-Cre*^*ERT2*^ driver strain. We then randomized *SMA-DTA* and *SMA-PPARγ*^*fl/fl*^ mice to vehicle or TM and to RT or cold temperature (Supplementary Fig. 8a). Vehicle-treated mice and mice lacking a salient allele (for example, no *DTA*) served as negative controls. Both strategies (*DTA* and *PPARγ*^*fl/fl*^) appeared to inhibit cold-induced beige adipocyte formation and appeared to alter the physiological responses to cold exposure (Fig. 8a–e). For example, the induced *SMA-DTA* and *SMA-PPARγ*^*fl/fl*^ mice had reduced body temperatures, elevated blood glucose levels, significant reductions in the quantities of multilocular or *UCP1*+ cells in haematoxylin and eosin and IHC, and disrupted induction of beige adipocyte/thermogenic genes; the levels approximated those of RT controls (Fig. 8a–e). The induced *SMA-DTA* and *SMA-PPARγ*^*fl/fl*^ mice had normal activity, body weight and food intake, and appeared phenotypically normal (Supplementary Fig. 8b,c, not shown)^14^. At RT, white and classical brown adipose depots and non-adipose tissues had normal weight and histological appearance, had normal vessels and did not display vascular leakage or haemorrhage, consistent with previous reports^14^ (Supplementary Fig. 8d–g). We also repeated the *Rosa26R-DTA* necessity studies using the *SMA-rtTA* driver strain and found similar results: lower temperature, elevated sera glucose and blunted beiging as assessed by histology and beige gene expression (Supplementary Fig. 9a–d). We also found that *SMA-rtTA-DTA* mutant mice housed at RT appeared normal and had preserved body weight and food intake (Supplementary Fig. 9e,f). To attempt to assess whether the blunted beiging observed *in vivo* might be due to cell autonomous actions, we turned to *ex vivo* analyses. For this, we TM induced *SMA-PPARγ*^*fl/fl*^ mice and then isolated and cultured SV cells in beige adipogenic conditions. Similar to the *in vivo* setting, beige adipocyte formation was disrupted with blunted expression of beige markers, indicating a potential cell autonomous effect (Fig. 8f).

A potential issue of deleting *PPARγ* in *SMA*+ mural cells could be changes in vascular function or integrity, which could alter cold responses and beiging. To pursue this notion we turned to the *Myh11-Cre*^*ERT2*^ mice, because in flow cytometry studies the *Myh11-Cre*^*ERT2*^*; RFP* marked roughly the same percentage (8–13%) of white adipose tissue vascular-residing mural cells as did the *SMA* drivers, and because *Myh11* is also expressed in many other mural compartments and muscle cells throughout the body (Supplementary Fig. 6). Nevertheless, the *Myh11-Cre*^*ERT2*^*; RFP* had a much lower beige adipocyte fate-mapping potential than *SMA* driver mice (Figs 1 and 2, and Supplementary Figs 1 and 2). Therefore, using the *Myh11-Cre*^*ERT2*^ driver to alter *PPARγ* function might allow discrimination between possible changes in vascular function, presumably present in roughly equal amounts in the *Myh11* and *SMA* driver settings. We incorporated the *PPARγ*^*fl/fl*^ allele with *Myh11-Cre*^*ERT2*^, to generate *Myh11-Cre*^*ERT2*^*; PPARγ* mice and induced *PPARγ* deletion by administering one dose of TM for 2 consecutive days. After washout, we housed these mice in the cold for 7 and 14 days, and our analyses indicated that cold-induced beiging and cold-induced physiological responses were unchanged. For example, body temperatures and sera glucose levels were similar between controls and mutants (Fig. 8g,h). Histological, IHC and molecular analyses indicated that beige adipocyte formation was unchanged between controls and mutants (Fig. 8i–k). *Myh11-Cre*^*ERT2*^*; PPARγ*-deleted mice appeared phenotypically normal and had similar body weight and adipose tissue weight at RT. We also did not observe any vasculature abnormalities or leak within the white adipose depots (not shown). We also analysed *ex vivo* beige adipogenic potential and isolated SV cells from TM-induced control and *Myh11-Cre*^*ERT2*^*; PPARγ* mice. We cultured the cells to confluence, incubated them in beige adipogenic conditions and quantified triglyceride accumulation and beige marker expression. These studies indicated that beige adipogenesis was relatively normal; both triglyceride content and mRNA levels of beige adipocyte markers were similar in control and mutant cells (Fig. 8l).

## Discussion

Cold-induced beige adipocytes convert glucose and fatty acids to heat, thereby potentially increasing energy expenditure^26^,^48^,^49^,^50^,^51^,^52^. These beige cellular furnaces could help address the widespread and conjoined obesity and diabetes epidemics^53^,^54^,^55^. Stimulating beige adipocyte formation and function may therefore lead to improved glucose homeostasis, increased metabolic rate and lower adiposity, and some clinical studies support this notion^56^,^57^,^58^,^59^. This therapeutic potential has been limited, because the origins of beige adipocytes are not well understood, because the precise cellular source of the beige phenomenon is controversial and because additional tools to mark and manipulate beige progenitors are needed.

Our data, based on a series of adipocyte, muscle and mural cell Cre and inducible Cre genetically modified mouse strains suggest that white adipose tissue-resident mural cells are an important cellular source of cold-induced beige adipocytes. Several mural-marking mice displayed fate mapping into beige adipocytes and two independent inducible *SMA*-based strains, *SMA-Cre*^*ERT2*^ and *SMA-rtTA*, appeared suited to explore and delineate cold-induced beige biology. For example, the majority of cold-induced beige adipocytes seemed to derive from the *SMA*-marked sources. Although neither white nor beige adipocytes appeared to express endogenous *SMA*, or *SMA-Cre*^*ERT2*^*-* or *SMA-rtTA-*driven RFP (on activation of the reporter), mural cells did and in a restricted manner. When we disrupted *SMA*-expressing cell function, by inhibiting adipocyte differentiation (deleting *PPARγ*) or with a cell-killing strategy (activating *DTA*) in *SMA-Cre*^*ERT2*^ cells and using *SMA-rtTA* to activate *DTA*, beiging was impaired *in vivo* and *ex vivo*. In these three mutant mouse strains, not only was beige adipocyte formation diminished but also the mutant mice were unable to appropriately defend body temperature and had elevated glucose levels. However, it is plausible that some of these effects on blood glucose and temperature could stem from roles of *SMA-Cre*^*ERT2*^- and SMA-rtTA-expressing cells in adipose mural cells that did not have progenitor potential and in non-adipose tissues such as cardiac and skeletal muscles, as both these drivers mark these tissues (Fig. 1 and Supplementary Figs 5 and 6)^21^. For example, the *SMA-DTA* models could have increased skeletal muscle cell death, which could lower body temperature due to impaired or lack of shivering (muscle contraction). Further, glucose uptake could also be diminished due to muscle atrophy. Nevertheless, histologically, these tissues appeared normal, the mice appeared healthy and had normal food intake and activity, and at RT the strains did not appear to have an altered phenotype. To attempt to control for some of these non-autonomous concerns, we also altered *PPARγ* function using the *Myh11-Cre*^*ERT2*^ driver; these mice had normal beiging, normal blood glucose levels and were able to defend their body temperature. Nonetheless, further characterization is needed to further delineate potential ‘side effects' of ablating *SMA*+ cells in all *SMA* driver-expressing tissues.

The data in this study in concert with other reports indicate that white and beige adipocyte progenitors may have common features such as lineage origin, cell compartment locality and others. For example, the *SMA* drivers' fate map into white adipocytes under the influence of some stimuli such as RT and into beige adipocytes under other conditions such as cold exposure. This raises an interesting scenario in which cold exposure first triggers the *SMA*-marked mural cells to undergo formation of white adipocytes that then convert to a beige adipocyte phenotype. This possibility is in line with the conventional hypothesis of a white to beige transdifferentiation. However, this is not yet clear whether the same *SMA*-marked cell can give rise to both white and beige adipocytes or to only one cell type. Further studies and genetic tools are needed to tease apart the functions and relationships of white and beige progenitors.

In addition to *SMA* we found that other mural cell Cre/inducible Cre drivers marked cold-induced beige adipocytes. In agreement with Spiegelman and colleagues^11^, we observed that *Myh11*+ cells fate mapped into 12% of beige adipocytes after 2 weeks of cold exposure. *AdipoTrak* also marked the cold-induced beiging process as did *NG2* and *SM22*; however, these tools have caveats. *AdipoTrak* in the unsuppressed state marks the mural progenitors, but also the forming adipose lineage and mature adipocytes. These issues can be overcome when combining Dox suppression/release and *H2B-GFP* marking, the labeling of actively proliferating cells in the mural compartment. With this method, we found that mural progenitor marking is relatively restricted but this restriction evolves under the influence of cold temperatures to include fate mapping into beige adipocytes. This supports the mural progenitor hypothesis and the notion that during lineage specification amplification of the compartment is present. *NG2-Cre*^*ERT2*^ has issues in subcutaneous depots, as it marked both the progenitor and adipocyte compartment, potentially masking the source of cold-induced beige adipocytes. Of note, in subcutaneous depots 100% of white adipocytes were marked, but only about 50% of beige adipocytes were marked after cold exposure, consistent with the possibility that non-adipocytes are key sources of cold-induced beiging. Similar outcomes (100% white adipocyte marking to 45% beige adipocyte marking post cold) in retroperitoneal depots of *Myf5-Cre; RFP* mice amplify this notion. In visceral depots, *NG2-Cre*^*ERT2*^ marking appeared to have relative restriction to the mural progenitor compartment, not marking white mature adipocytes, and did fate map into cold-induced beige adipocyte formation. The *SM22-Cre* strain labelled a majority of beige adipocytes and the mural perivascular compartment; however, this tool suffers from the lack of inducibility of straight Cre tool/systems; active and historically *SM22-Cre*-expressing cells will be labelled. This cell marking system is likely to be inappropriate for fate mapping because of the inability to determine when and where *SM22* is expressed (stem, progenitor, mature adipocyte and embryonic timing and so on).

The fact that a wide range of independently derived mural marking strains (for example, *AdipoTrak*, *SM22-Cre*, *NG2-Cre*^*ERT2*^, *Myh11-Cre*^*ERT2*^, *SMA-Cre*^*ERT2*^ and *SMA-rtTA*) fate map into cold-induced beiging indicates that mural cells are a beige progenitor source. However, the strains were expressed in common and independent sets of adipose depot mural cells and the endogenous proteins also appeared to label distinct populations of mural cells. This heterogeneity may in part account for why the drivers had different fate-mapping efficiencies. Potential insight into the relationship of the different murals may be gleaned from the increase in beige adipocyte marking percentage of the *Myh11* strain when comparing 1 week with 2 weeks of cold exposure. This may potentially identify an evolution of the marked cells during this time frame. More extensive lineage studies are needed to tease apart the relationship between the various mural markers and the beige progenitor mural marker signature. The development of new genetic tools may foster the ability to dissect the relevant cell types and specification steps that underlie formation of beige adipocytes.

Taken together, studies support the notion that mural cells are an important source of cold-induced beiging, and that inducible *SMA*-based engineered mice, *SMA-Cre*^*ERT2*^ or *SMA-rtTA*, are appropriate strains to study cold-induced beige adipocyte formation and function^8^,^11^,^51^,^52^. These mouse strains and derivatives thereof may enable identification and isolation of the beige progenitors and their descendants, as well as delineation of signals and mechanisms that control cold-inducible beige behaviour—essential steps on the road to therapeutic manipulation.

## Methods

### Animals

All animals were maintained under the ethical guidelines of the UT Southwestern Medical Center Animal Care and Use Committee according to NIH guidelines. Mice were housed in a 12:12 light:dark cycle at 23 °C, and chow and water were provided *ad libitum*. *Adiponectin-Cre*^*ERT2*^ (Stock 024671), *Myf5-Cre* (Stock 007893), *SM22-Cre* (Stock 017491), *Myh11-Cre*^*ERT2*^ (Stock 019079), *NG2-Cre*^*ERT2*^ (Stock 008538), *PPARγ*^*fl/fl*^ (Stock 004584), *Rosa26R*^*DTA*^ (Stock 006331) and *Rosa26R*^*RFP*^ (Stock 007914) mice were obtained from the Jackson Laboratory. AdipoTrak and aP2-Cre^ERT2^ were previously described^11^. Dr Eric Olson generously provided *Myogenin-Cre* and *UCP1-Cre*^*ERT2*^ mice. Dr Pierre Chambon generously provided *SMA-Cre*^*ERT2*^ mice. Dr Beverly Rothermel generously provided *SMA-rtTA mice*. Drs Sean Morrison and Bill Richardson generously provided the *PDGFRα-Cre*^*ERT2*^ mice. We performed experiments on 2-month-old males, unless specific ages were specified. All Cre mouse strains were maintained on a C57BL/6J and 129SV mixed background. Pure C57BL/6J 2-month-old male mice were purchased from Jackson Laboratories. Cre recombination was induced by administering TM dissolved in sunflower oil (Sigma, 100 mg kg^−1^ interperitoneal injection) on 2 consecutive days. Twenty-four hours post TM injection is considered a pulse. rtTA activation was induced by Dox (0.5 mg ml^−1^ in 1% sucrose) provided in the drinking water and protected from light, and it was changed every 2–3 days. For cold studies, mice were placed in a 6 °C cold metabolic chamber for 7–14 days. After recombination was induced, mice were randomized to RT or cold exposed for 7–14 days. We performed experiments on mice after a 48-h TM washout period, a week TM washout period and a 2-week TM washout period. No animals were excluded from any experiments unless mice displayed wounds from fighting.

### Histology

Haematoxylin and eosin staining was carried out on paraffin sections using standard methods. Adipose tissues were fixed in formalin overnight, dehydrated, embedded in paraffin and sectioned with a microtome at 5–8 μm thicknesses. Immunostaining was performed in either paraffin sections or cryostat sections (5–8 μm) of tissues freshly embedded in optical coherence tomography^13^. Briefly, samples were pre-incubated with permeabilization buffer (0.3% Triton X-100 in PBS) for 30 min at RT and then incubated sequentially with primary antibody (4 °C, overnight) and secondary antibody (2 h at RT), all in blocking buffer (5% normal donkey serum in 1 × PBS). Antibodies used for immunostaining are as follows: rabbit-anti-UCP1 (1:200, Abcam), mouse-anti-RFP (1:100, Clontech), rat-anti-PECAM (1:200, BD Biosciences), rabbit-anti-α-SMA (1:200, Sigma) and goat-anti-Perilipin (1:200, BD Biosciences). For negative control subjects, primary antibody was replaced with 5% normal donkey serum. Secondary antibodies including cy3 donkey anti-mouse, cy2 donkey anti-rabbit and cy2 donkey anti-rat were from Jackson ImmunoResearch. All secondary antibodies were used at a 1:500 dilution. Lipid was stained with LipidTOX Green (1:100, Life Technologies). Immunostaining images were collected on a Zeiss LSM500 confocal microscope, an Olympus IX70 inverted microscope or an Olympus upright BX40 microscope. All experimental and control images of the immunofluorescence data were collected using identical imaging settings. Paraffin-embedded tissues were sectioned with a Microm HM 325 microtome. Cryostat sectioning was performed with a Microm HM505 E cryostat.

### Co-localization quantification

For quantification of images, two independent observers assessed three random fields in ten random sections from at least three mice per cohort. NIH Image J software using the JACoP^60^ (Just Another Colocalization Plugin) extension was used to quantify co-localization. First, the total area of UCP1 immunostaining was calculated. Next, the area of co-localized UCP1 immunostaining and RFP (for all drivers) was calculated. To derive the total percentage of co-localization, the per cent co-localization between UCP1 immunostaining and RFP was divided by the total area of UCP1 immunostaining. To identify SMA-Cre^ERT2^; RFP expression in mature adipocytes, mice were administered one dose of TM for 2 consecutive days. Subcutaneous and visceral adipose depots were isolated and adipocytes were fractioned by floatation. Adipocytes were stained with LipidTOX (Life Technologies) and DAPI (4,6-diamidino-2-phenylindole) to visualize intact adipocytes. For quantification of images, two independent observers assessed three random fields in ten random sections from at least three mice per cohort. Positivity was assessed by LipidTox+, DAPI+ and RFP+.

### Stromal vascular fractionation

The SV cells were isolated^13^. Briefly, we pooled subcutaneous (inguinal and periscapular) white adipose tissues for fractionation, unless indicated otherwise. After 2 h of slow shaking in isolation buffer (100 mM HEPES pH 7.4, 120 mM NaCl, 50 mM KCl, 5 mM glucose, 1 mM CaCl2, 1.5% BSA) containing 1 mg ml^−1^ collagenase at 37 °C, the digest was pipetted up and down a few times for better dissociation. The suspension was then spun at 800*g* for 10 min; the resultant floating layer was the adipocyte layer and the pellet was a crude SV fraction. The floating adipocyte layer was washed in 1 × PBS, spun at 800*g* for 5 min and the solution was removed from below. The pellet was then resuspended in erythrocyte lysis buffer (0.83% NH_4_Cl in H_2_O) for 8 min and spun at 800*g* for 5 min. The pellet was washed once in 1 × PBS, resuspended and passed through 30 μm mesh.

### Cell culture

Isolated SV cells were cultured in DMEM supplemented with 10% fetal bovine serum (FBS). White adipogenesis was induced by treating confluent cells with DMEM containing 10% FBS, insulin (0.5 μg ml^−1^), dexamethasone (5 μM) and isobutylmethylxanthine (0.5 mM)^11^. Beige adipogenesis was induced by treating confluent cells with DMEM containing 10% FBS, insulin (0.5 μg ml^−1^), dexamethasone (5 μM), isobutylmethylxanthine (0.5 mM), troglitazone (1 μM), 5 nM indomethacin and T3 (2 nM)^44^. To induce thermogenic genes, cells were treated with 10 μM forskolin for 4 h and harvested to collect mRNA^55^. Triglyceride accumulation assay was performed using a kit from ZenBio^61^ following the manufacturer's protocol. To induce *SMA-Cre*^*ERT2*^*; RFP* recombination *ex vivo*, adipocytes were isolated from 2-month-old uninduced *SMA-Cre*^*ERT2*^*; RFP* male mice. TM (1 μM) was then administered *ex vivo* for 24 h and the floated adipocytes were fluorescence microscopically assessed for RFP and lipid (LipidTox, Life Technologies).

### Flow cytometry and sorting

SV cells were isolated^11^ and washed, centrifuged at 800*g* for 5 min and analysed with a FACScans analyser or sorted with a BD FACS Aria operated by the UT Southwestern Flow Cytometry Core. Data analysis was performed using BD FACS Diva software. For RFP+ sorting, live SV cells from young 2-month-old *R26R*^*RFP*^ mice were stained with propidium iodide (1 mg ml^−1^) to exclude dead cells and sorted based on native fluorescence (RFP). The SV cells from control mice were used to determine background fluorescence levels. The dissociated SV cells were also analysed for mural and perivascular markers by flow cytometry. Briefly, SV cells were incubated with primary antibodies on ice for 30 min and then washed twice with the staining buffer and incubated with secondary antibody for another 30 min on ice before flow cytometric analysis.

### Quantitative real-time PCR

Total RNA was extracted using TRIzol (Invitrogen) from either mouse tissues or cells. Complementary DNA synthesis was performed using Superscript III reverse transcriptase (Invitrogen) using 1 μg of RNA^13^. Gene expression was analysed using Power SYBR Green PCR Master Mix with ABI 7500 Real-Time PCR System. qPCR values were normalized by 18s rRNA expression. Primer sequences are available on request.

### Metabolic phenotyping experiments

Temperature was monitored daily using a rectal probe (Physitemp)^62^. Briefly, the probe was lubricated with glycerol and was inserted 1.27 cm (1/2 inch) and temperature was measured when stabilized. Glucose monitoring, that is, blood glucose levels were measured with a Contour glucometer (Bayer).

### Statistical analysis

Statistical significance assessed by two-tailed Student's *t*-test. Data are shown as means and error bars indicate s.e.m. Studies were performed on two or three independent cohorts and were performed on three to four mice per group unless specified. Sample size was determined using previous experimental studies for fate mapping and metabolic assessment. Mice were randomized to the cold temperature or RT in a blinded manner.

## Additional information

**How to cite this article:** Berry, D. C. *et al.* Mouse strains to study cold-inducible beige progenitors and beige adipocyte formation and function. *Nat. Commun.* 7:10184 doi: 10.1038/ncomms10184 (2016).

## Supplementary Material

Supplementary Supplementary Figures 1-9
