# Supplementary material for: Mouse strains to study cold-inducible beige progenitors and beige adipocyte formation and function
Source: Nat Commun. 2016 Jan 5;7:10184. doi: 10.1038/ncomms10184 (PMC4728429; doi:10.1038/ncomms10184)
Supplement: Supplementary — Figures 1-9 [file ncomms10184-s1.pdf]

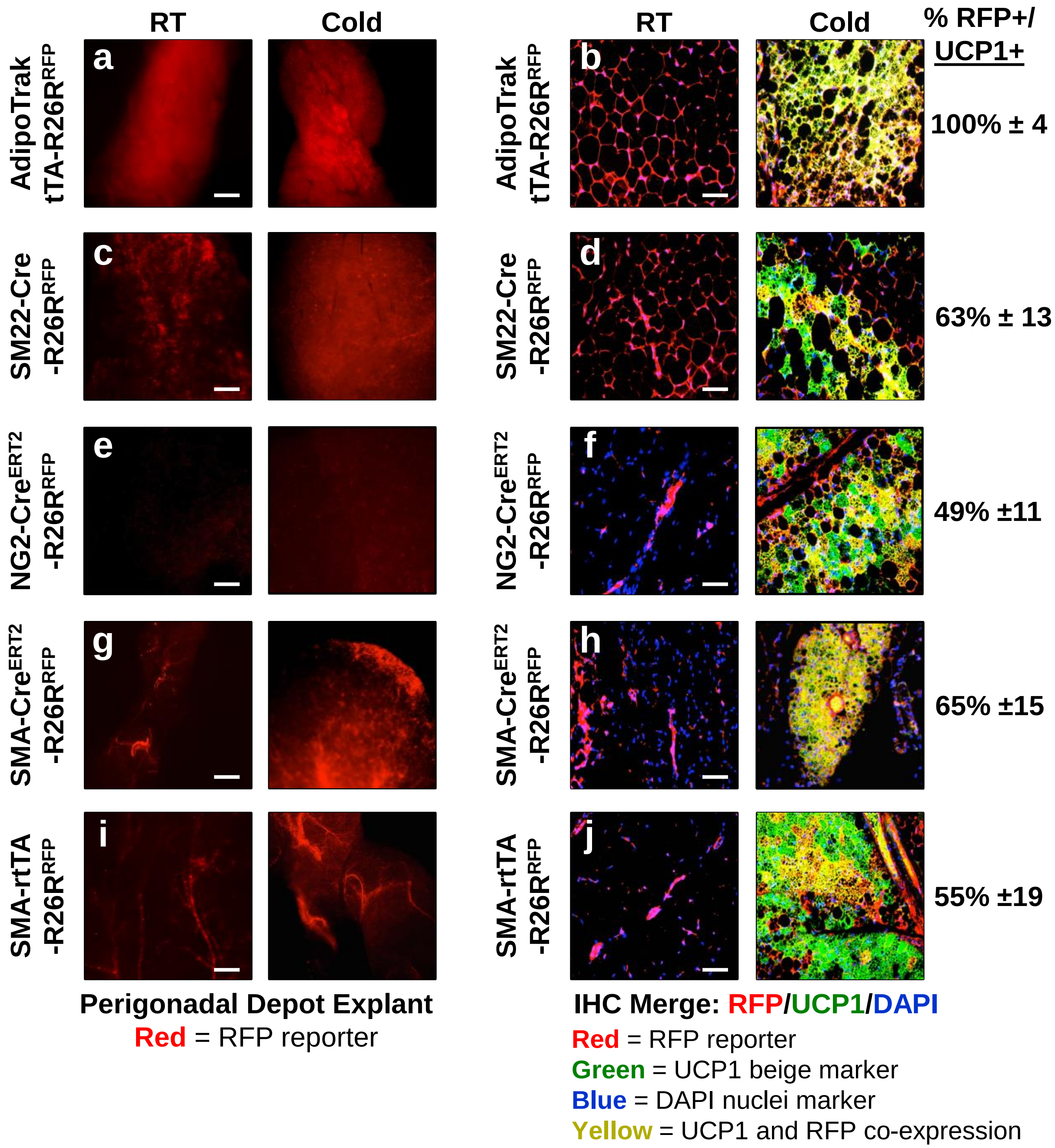

Supplementary Fig. 1

a-d) Two-month-old Cre (AdipoTrak and SM22); *R26R*^{RFP} mice were maintained at room temperature (23°C) or cold exposed (6°C) for seven days. Perigonadal adipose depot explants were imaged for direct RFP fluorescence or sectioned and immunostained for reporter (RFP) and UCP1 (green); co-expression (RFP + UCP1) is a yellow-gold hue. Cell nuclei were counterstained with DAPI (blue). Scale bar represents 10 μM. **e-j**) Two-month-old inducible Cre (*NG2-Cre*^{ERT2}*; SMA-Cre*^{ERT2} and *SMA-rtTA-*

TRE-Cre); *R26R*^{RFP} mouse models were administered one dose of tamoxifen on two consecutive days or administered doxycycline 3 days prior to experimentation. Mice were maintained at room temperature or cold exposed for seven days. Perigonadal adipose depot explants were imaged for direct RFP fluorescence or sectioned and immunostained for reporter (RFP) and UCP1 (green); co-expression (RFP + UCP1) is a yellow-gold hue. Images are representative from n = 4 mice/group replicated 2 times. Scale bar represents 10 μm (**a**, **c**, **e**, **g** and **i**). Scale bar represents 100 μm (**b**, **d**, **f**, **h** and **j**).

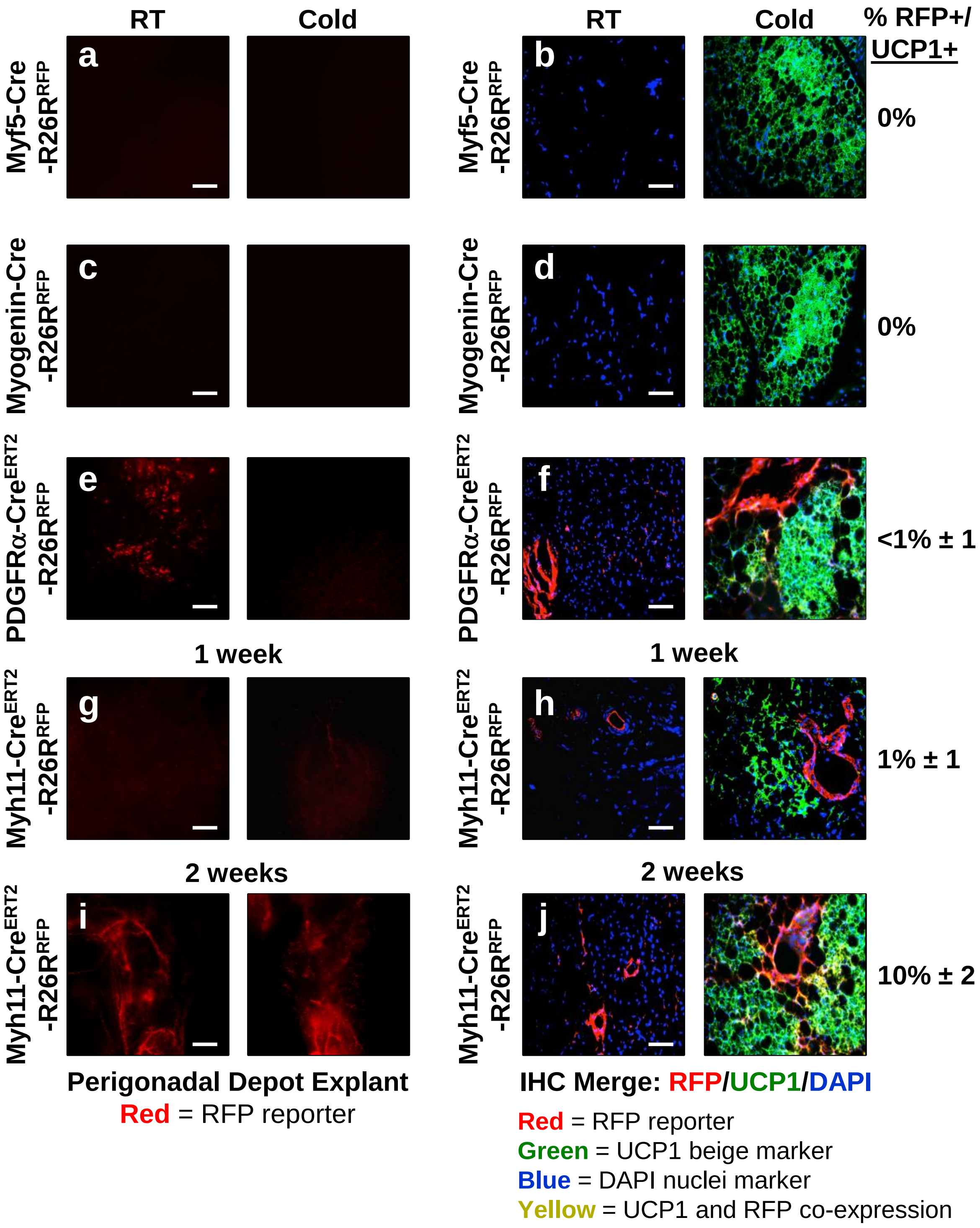

Supplementary Fig. 2

a-d) Two-month-old Cre (*Myf5-Cre* and *Myogenin (MyoG)-Cre*); *R26R^{RFP}* mice were maintained at room temperature (23°C) or cold exposed (6°C) for seven days. Perigonadal adipose depot explants were imaged for direct RFP fluorescence or sectioned and immunostained for reporter (RFP) and UCP1 (green). Scale bar

represents 10 μM. **e-h**) Two-month-old inducible Cre (*PDGFR*α*-Cre*^{ERT2} and *Myh11-Cre*^{ERT2}); *R26R*^{RFP} mice were administered one dose of tamoxifen on two consecutive days. Mice were maintained at room temperature or cold exposed for seven days. Perigonadal adipose depot explants were imaged or sectioned and immunostained for reporter (RFP) and UCP1 (green). Cell nuclei were counterstained with DAPI (blue). **i, j**) *Myh11-Cre*^{ERT2}*; RFP* mice were administered one dose of tamoxifen on two consecutive days. Mice were maintained at room temperature or cold exposed for fourteen days. Images are representative from n = 4 mice/group replicated 2 times. Scale bar represents 10 μm (**a**, **c**, **e**, **g** and **i**). Scale bar represents 100 μm (**b**, **d**, **f**, **h** and **j**).

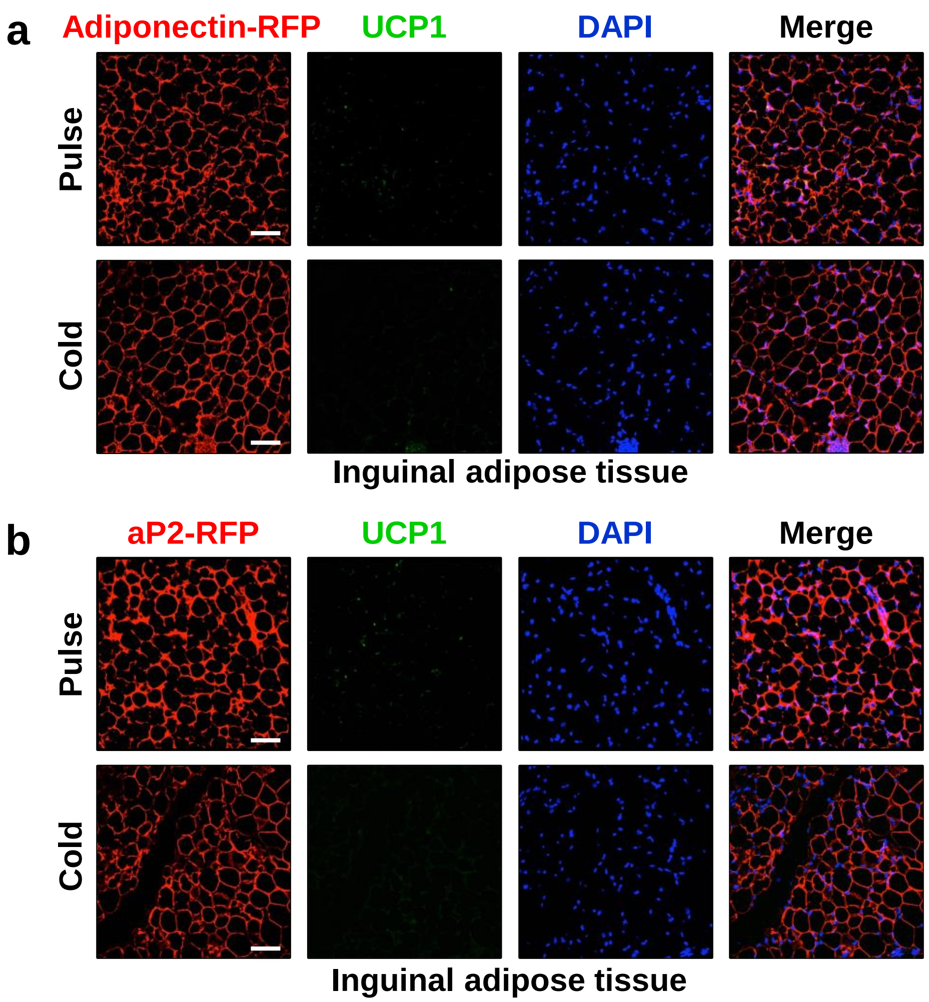

Supplementary Fig. 3

a, b) Two-month-old *Adiponectin-Cre^{ERT2}; RFP* (**a**) and *aP2-Cre^{ERT2}: RFP* (**b**) male mice were administered one dose of TM for two consecutive days and examined (pulse) or mice were maintained at room temperature for seven days (TM washout period). Subsequently mice were cold exposed for seven days. Subcutaneous inguinal adipose depots were sectioned and immunostained for RFP (red) and UCP1 (green). Cell nuclei were visualized by DAPI staining. These images focus on areas that contain white adipocytes only, highlighting that cold exposed white adipocytes do not express UCP1 by UCP immunostaining. Images are representative from n = 3 mice/group replicated twice Scale bar represents 100 µm.

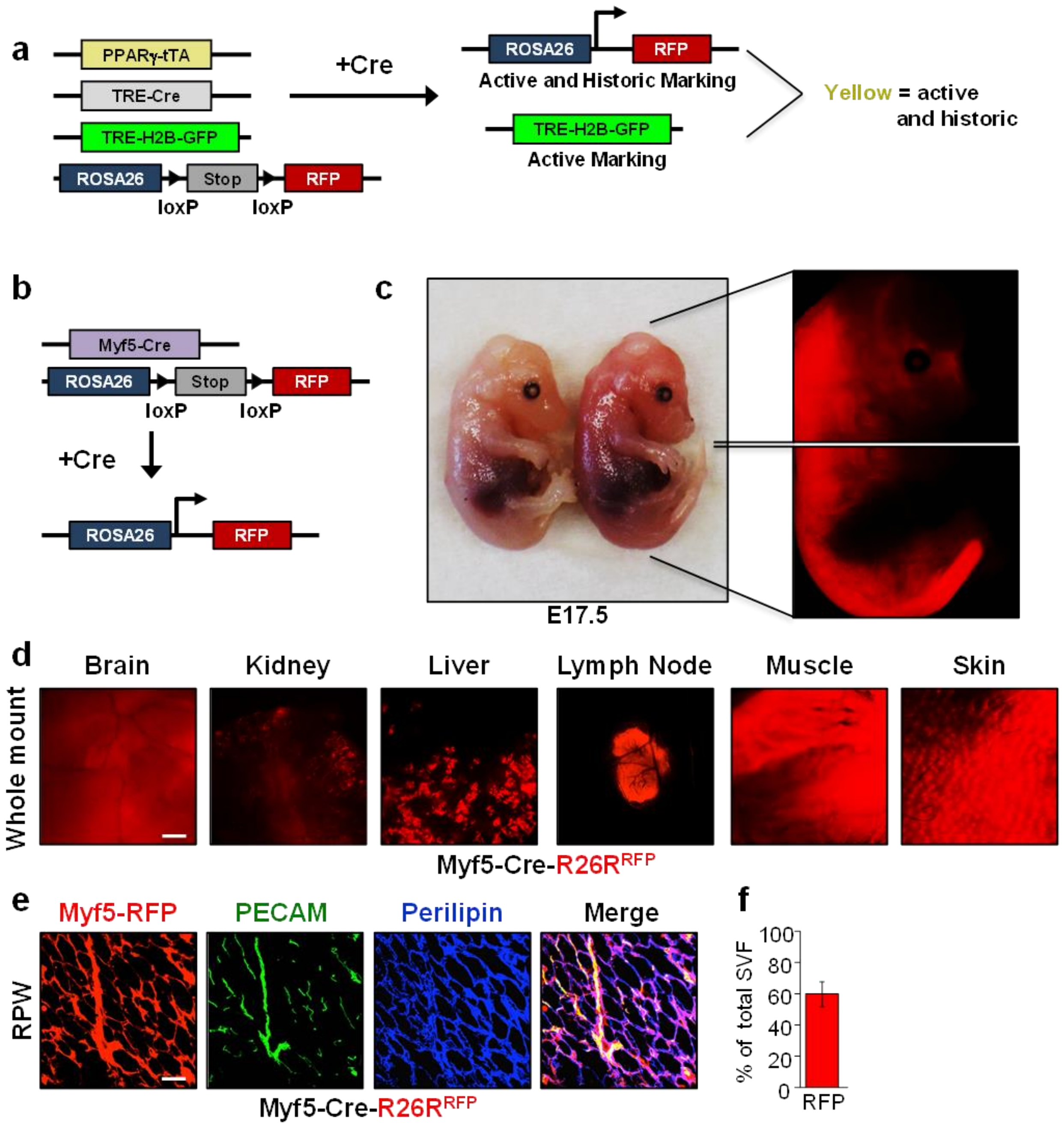

Supplementary Fig. 4

a) Genetic alleles denoting the *AdipoTrak (PPAR*γ^{tTA}*); TRE-H2B-GFP; R26R*^{RFP} mice. **b**) Genetic alleles denoting the *Myf5-Cre; R26R*^{RFP} (*Myf5-Cre; RFP*) mice. **c**) Photograph and RFP fluorescence imaging of control (*R26R*^{RFP}) and *Myf5-Cre; RFP* E17.5 embryo. **d**) Whole mount RFP fluorescent images of denoted tissues from two-month-old *Myf5-Cre; RFP* mice. Scale bar represents 10 μM. **e**) Immunostaining of RFP, PECAM and perilipin from retroperitoneal (RPW) adipose depots from two-month-old *Myf5-Cre; RFP*

mice. **f**) SV cells were isolated from the periscapular and retroperitoneal white adipose depots from two-month-old room temperature *Myf5-Cre; RFP* mice and analyzed for expression of RFP using flow cytometry and the data that about 60% of the SV cells were *Myf5-Cre; RFP* positive. Data are means ± s.e.m (n = 4-5 mice/group); *P-value <0.05 RFP+ compared to RFP-. Scale bar represents 100 μm.

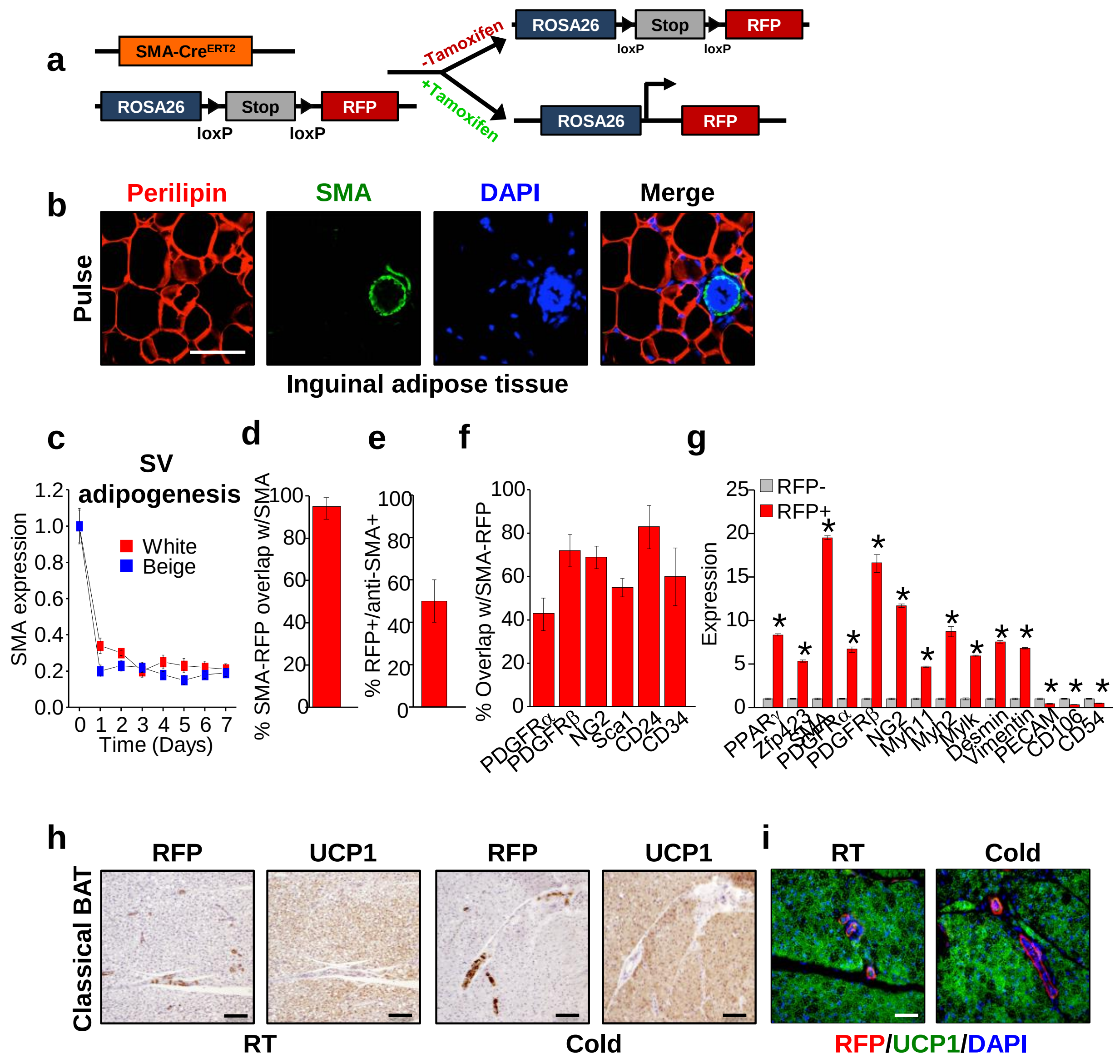

Supplementary Fig. 5

a) Genetic alleles denoting the *SMA-Cre*^{ERT2}*; R26R*^{RFP} mice. **b**) Two-month-old *SMA-Cre*^{ERT2}*; R26R*^{RFP} male mice were administered one dose of TM for two consecutive days. 48 hours after the last injection subcutaneous inguinal adipose depots were sectioned and stained for Perilipin (red) and endogenous SMA (green). Cell nuclei were visulalized by DAPI. **c**) SV cells were isolated from two-month old wild-type mice. Cells were induced with white or beige adipogenic conditions and SMA mRNA expression was analyzed every day for seven days by qPCR. **d-f**) SV cells were isolated from two-

month-old *SMA-Cre*^{ERT2}*; R26R*^{RFP} pulsed male mice. SV cells were analyzed by flow cytometry for RFP (**d**), SMA (**e**) and indicated mural and progenitor genes (**f**). **g**) SV cells from two-month-old pulse *SMA-Cre*^{ERT2}*; R26R*^{RFP} mice were separated into RFP+ and RFP- fractions using FACS and examined for mRNA expression of mural, endothelial and adipose progenitor markers. Data are means ± s.e.m (n = 4-5 mice/group); *P-value <0.05 RFP+ compared to RFP-. **h,i**) Interscapular brown adipose tissue was isolated from two-month-old *SMA-Cre*^{ERT2}*; R26R*^{RFP} male mice exposed to room temperature or cold for seven days. IHC or immunostaining (merged images) for RFP and UCP1 was performed. Scale bar represents 100 μm.

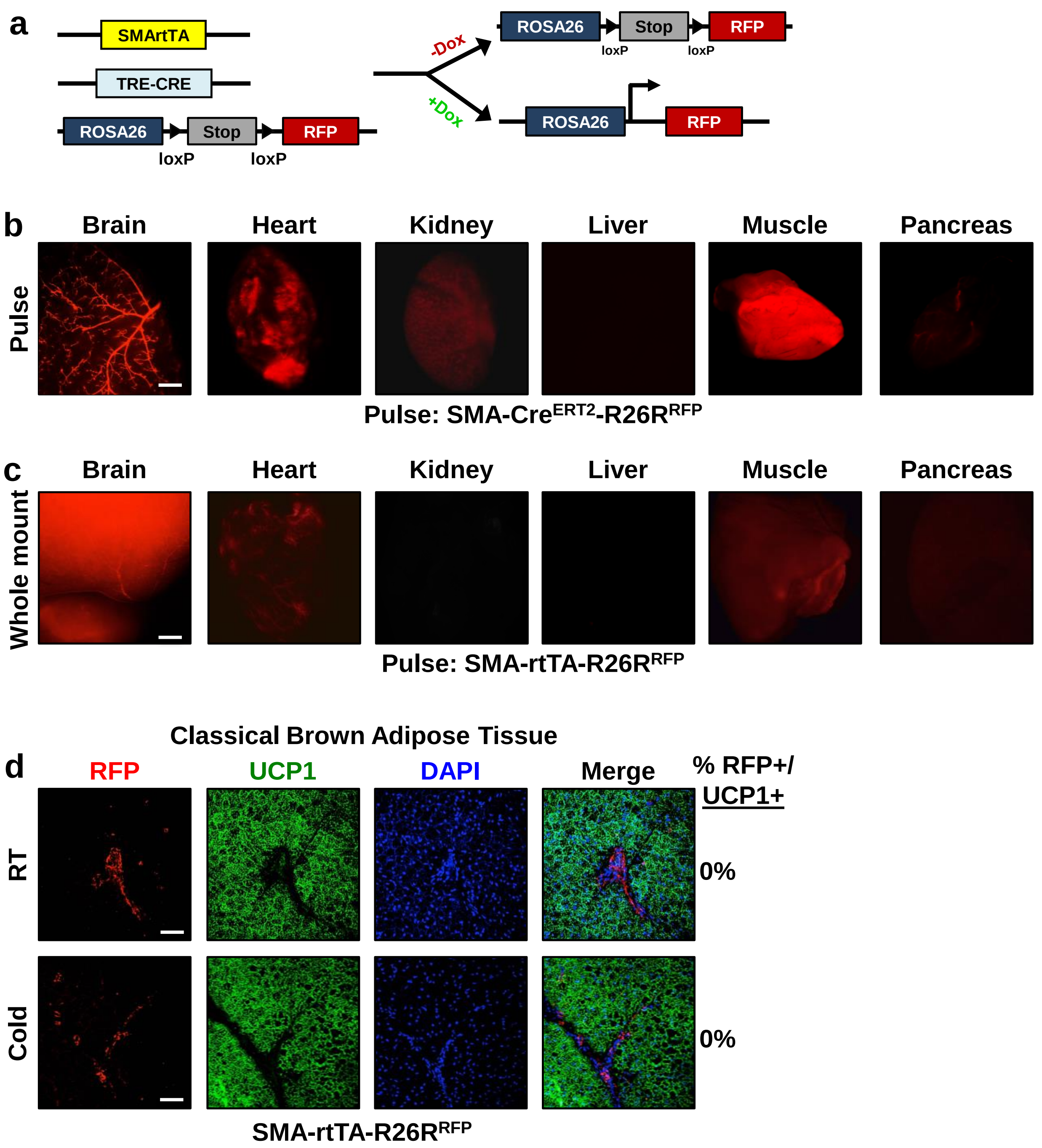

Supplementary Fig. 6

a) Genetic alleles denoting the *SMA-rtTA; TRE-Cre; R26R*^{RFP} (*SMA-rtTA; RFP*) mouse model. **b, c**) Whole mount RFP fluorescent images of denoted organs from *SMA-Cre*^{ERT2}*; R26R*^{RFP} (**b**) or *SMA-rtTA; RFP* (**c**) mice. Scale bar represents 10 μM. **d**) Interscapular brown adipose tissue was isolated from two-month-old *SMA-Cre*^{ERT2}*;*

R26R^{RFP} male mice exposed to room temperature or cold for seven days.

Immunostaining for RFP and UCP1 was performed. Scale bar represents 100 μm.

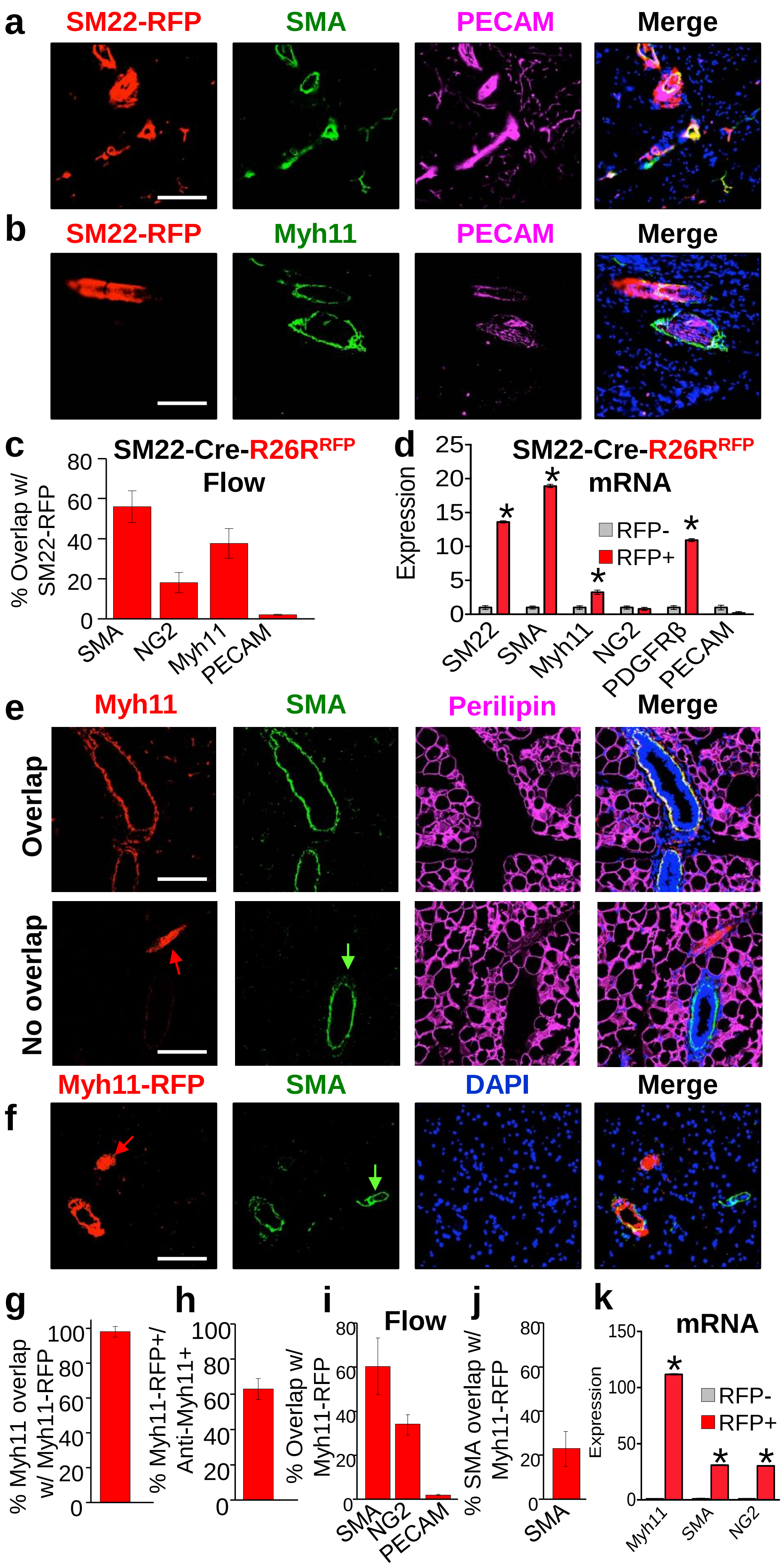
a
SM22-RFP
SMA
PECAM
Merge
b
SM22-RFP
Myh11
PECAM
Merge
c
SM22-Cre-R26R^RFP
Flow
% Overlap w/ SM22-RFP
SMA
NG2
Myh11
PECAM
d
SM22-Cre-R26R^RFP
mRNA
Expression
RFP-
RFP+
SM22
SMA
Myh11
NG2
PDGFRβ
PECAM
e
Myh11
SMA
Perilipin
Merge
Overlap
No overlap
f
Myh11-RFP
SMA
DAPI
Merge
g
% Myh11 overlap w/ Myh11-RFP
h
% Myh11-RFP+/ Anti-Myh11+
i
Flow
% Overlap w/ Myh11-RFP
SMA
NG2
PECAM
j
% SMA overlap w/ Myh11-RFP
SMA
k
mRNA
Expression
RFP-
RFP+
Myh11
SMA
NG2

Supplementary Fig. 7

a, b) Subcutaneous inguinal adipose depots from two-month-old SM22-Cre; RFP male mice were sectioned and immunostained for RFP, SMA (**a**) or Myh11 (**b**), and PECAM. Cell nuclei were visualized using DAPI. **c**) SM22/RFP+ cells were FACS isolated from two-month-old SM22-Cre; RFP male mice. SM22/RFP cells were analyzed by flow for co-expression of indicated genes with RFP. **d**) Two-month-old Myh11-Cre^{ERT2}; RFP male mice were administered one dose of TM for two consecutive days. 48 hours after the last injection subcutaneous inguinal adipose depots were sectioned and stained for RFP and SMA. Cell nuclei were visualized with DAPI. **e**) Subcutaneous adipose depots from wild-type mice were immunostained for Myh11, SMA and perilipin. In some sections there is overlap (top) between anti-Myh11 and anti-SMA but in some sections there is not (below). **f**) Subcutaneous inguinal adipose depots from TM pulsed *Myh11-*Cre^{ERT2}*; *$R26R^{RFP}$ mice were immunostained for RFP and SMA. **g, h**) SV cells were isolated from two-month-old *Myh11-*Cre^{ERT2}*; *$R26R^{RFP}$ pulsed male mice. SV cells were analyzed by flow cytometry for Myh11 overlap with RFP (**g**) and RFP overlap with total anti-Myh11 (**h**). Cells in (**g**) were examined for RFP overlap with SMA, NG2 and PECAM. **j**) SV cells were isolated from two-month-old *Myh11-*Cre^{ERT2}*; *$R26R^{RFP}$ pulsed male mice. SV cells were stained with anti-SMA and analyzed by flow cytometry for RFP overlap. **k**) SV cells were isolated from two-month-old *Myh11-*Cre^{ERT2}*; *$R26R^{RFP}$ male mice. Cells were FACS sorted into RFP- and RFP+ and were analyzed for Myh11, SMA and NG2 mRNA expression (**g**). Data are means ± s.e.m (n = 4-5 mice/group); *P-value <0.05 RFP+ compared to RFP-. Scale bar represents 100 µm.

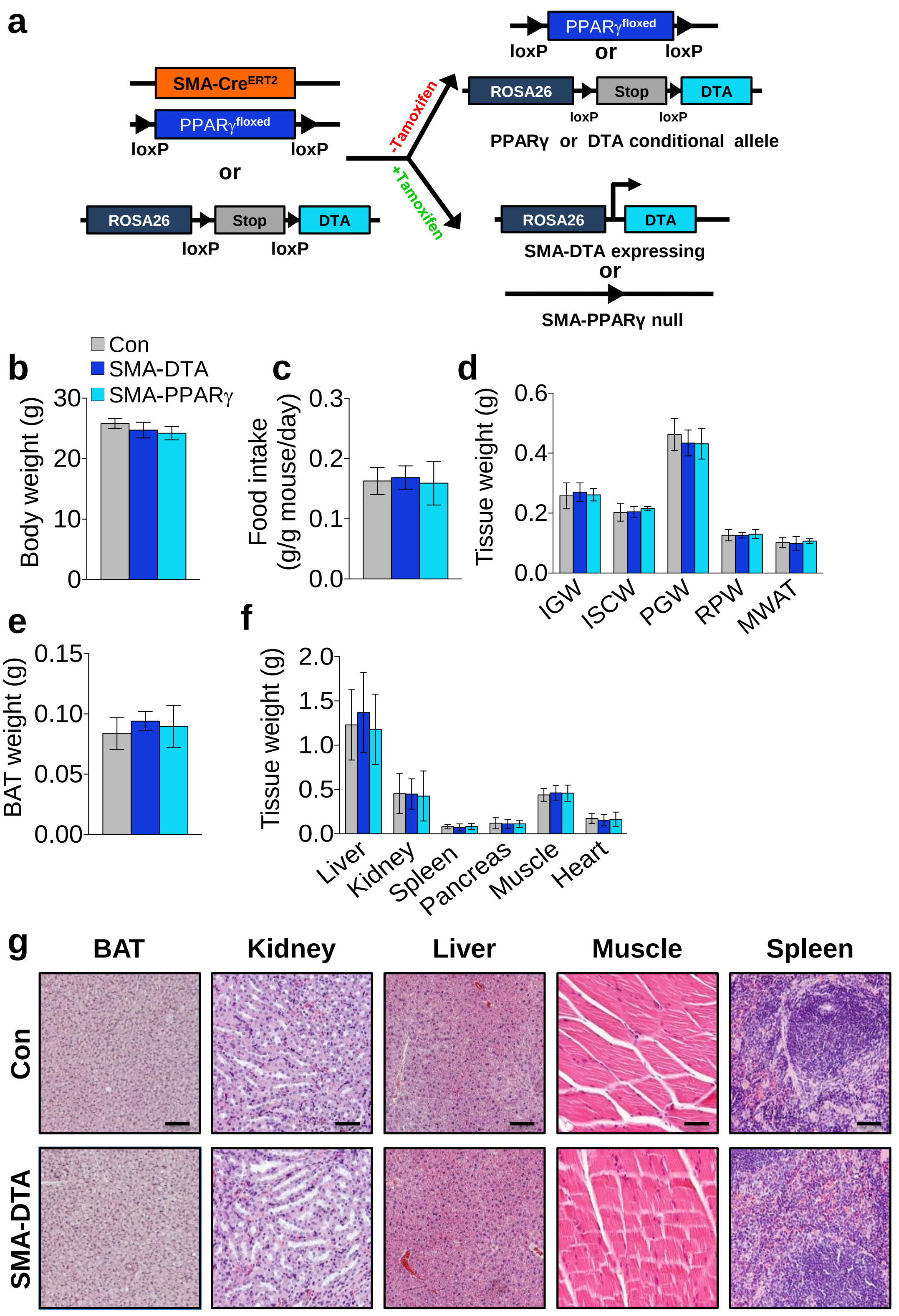
a
SMA-Cre^ERT2
PPARγ^floxed
loxP
loxP
or
ROSA26
Stop
DTA
loxP
loxP
-Tamoxifen
+Tamoxifen
PPARγ^floxed
loxP
or
loxP
ROSA26
Stop
DTA
loxP
loxP
PPARγ or DTA conditional allele
ROSA26
DTA
SMA-DTA expressing
or
SMA-PPARγ null
b
Con
SMA-DTA
SMA-PPARγ
Body weight (g)
30
20
10
0
c
Food intake (g/g mouse/day)
0.3
0.2
0.1
0.0
d
Tissue weight (g)
0.6
0.4
0.2
0.0
IGW
ISCW
PGW
RPW
MWAT
e
BAT weight (g)
0.15
0.10
0.05
0.00
f
Tissue weight (g)
2.0
1.5
1.0
0.5
0.0
Liver
Kidney
Spleen
Pancreas
Muscle
Heart
g
BAT
Kidney
Liver
Muscle
Spleen
Con
SMA-DTA

Supplementary Fig. 8

a) Genetic alleles denoting the *SMA-Cre*^{ERT2}*; R26R-DTA* or *PPAR*$\gamma^{fl/fl}$ mice. **b-f**) Mice described in (**a**) were administered one dose of tamoxifen on two consecutive days and mice were maintained at room temperature for seven days. Body weight (**b**), food intake (**c**), adipose tissue weights (**d**), classical brown adipose tissue (BAT) weight (**e**) and other organ weights (**f**) were measured. **g**) H&E staining of denoted tissues from *SMA-Cre*^{ERT2}*; R26R-DTA* or control mice described in (**a**). Scale bar represents 100 μm.

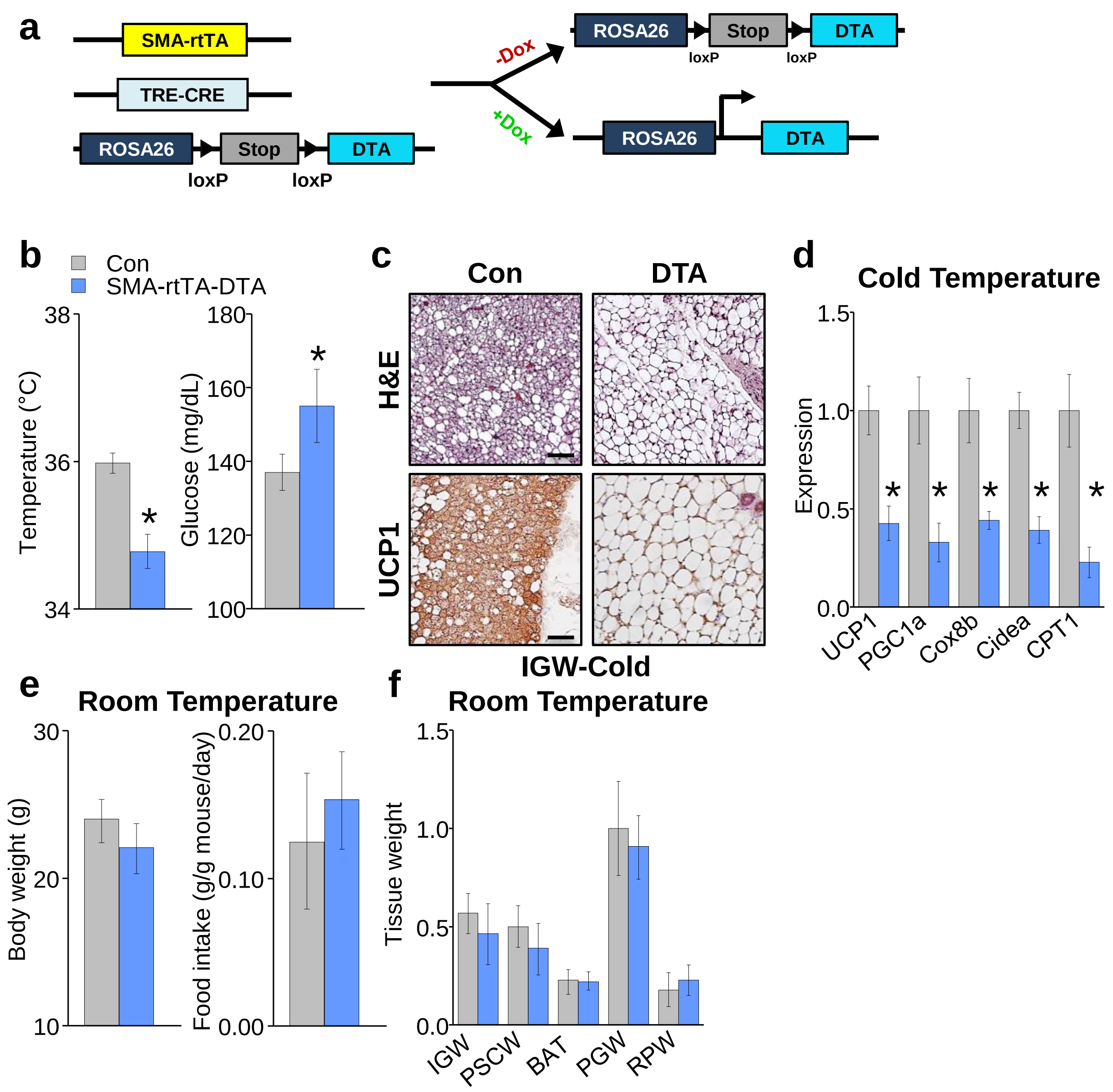

Supplementary Fig. 9

a) Genetic alleles denoting the *SMArtTA;* TRE-Cre and *R26R-DTA* mice. **b-f**) Mice described in (**a**) were administered doxycycline three days prior to cold exposure and subsequently exposed to cold for seven days. Beiging was assessed by: rectal temperature and sera glucose (**b**), H&E and UCP1 IHC staining (**c**), and beige gene expression (**d**). **e**) Mice described in (**a**) were assessed for body weight, food intake and adipose tissue weight at room temperature . Data are means ± s.e.m (n = 4-5 mice/group); *P-value <0.05 mutant compared to control. Scale bar represents 100 μm.
